# JNK Mediates Differentiation, Cell Polarity and Apoptosis During Amphioxus Development by Regulating Actin Cytoskeleton Dynamics and ERK Signalling

**DOI:** 10.3389/fcell.2021.749806

**Published:** 2021-10-29

**Authors:** Ildiko M. L. Somorjai, Matthias T. Ehebauer, Hector Escrivà, Jordi Garcia-Fernàndez

**Affiliations:** ^1^School of Biology, University of St Andrews, St Andrews, United Kingdom; ^2^Sorbonne Université, CNRS, Biologie Intégrative des Organismes Marins, Observatoire Océanologique, Banyuls-sur-Mer, France; ^3^Departament de Genètica, Microbiologia i Estadística, University of Barcelona, Barcelona, Spain; ^4^European Molecular Biology Laboratory, Hamburg Unit, Hamburg, Germany; ^5^Institut de Biomedicina, University of Barcelona, Barcelona, Spain

**Keywords:** JNK, apoptosis, amphioxus, chordate, ERK, cellular extrusion, Wnt, notochord

## Abstract

c-Jun N-terminal kinase (JNK) is a multi-functional protein involved in a diverse array of context-dependent processes, including apoptosis, cell cycle regulation, adhesion, and differentiation. It is integral to several signalling cascades, notably downstream of non-canonical Wnt and mitogen activated protein kinase (MAPK) signalling pathways. As such, it is a key regulator of cellular behaviour and patterning during embryonic development across the animal kingdom. The cephalochordate amphioxus is an invertebrate chordate model system straddling the invertebrate to vertebrate transition and is thus ideally suited for comparative studies of morphogenesis. However, next to nothing is known about JNK signalling or cellular processes in this lineage. Pharmacological inhibition of JNK signalling using SP600125 during embryonic development arrests gastrula invagination and causes convergence extension-like defects in axial elongation, particularly of the notochord. Pharynx formation and anterior oral mesoderm derivatives like the preoral pit are also affected. This is accompanied by tissue-specific transcriptional changes, including reduced expression of *six3/6* and *wnt2* in the notochord, and ectopic *wnt11* in neurulating embryos treated at late gastrula stages. Cellular delamination results in accumulation of cells in the gut cavity and a dorsal fin-like protrusion, followed by secondary Caspase-3-mediated apoptosis of polarity-deficient cells, a phenotype only partly rescued by co-culture with the pan-Caspase inhibitor Z-VAD-fmk. Ectopic activation of extracellular signal regulated kinase (ERK) signalling in the neighbours of extruded notochord and neural cells, possibly due to altered adhesive and tensile properties, as well as defects in cellular migration, may explain some phenotypes caused by JNK inhibition. Overall, this study supports conserved functions of JNK signalling in mediating the complex balance between cell survival, apoptosis, differentiation, and cell fate specification during cephalochordate morphogenesis.

## Introduction

Embryogenesis requires the accurate temporal and spatial coordination of tissue fate, cell movements, and cell numbers in a controlled and reproducible way. In most metazoans, this is achieved by the complex orchestration of only seven evolutionarily conserved developmental pathways, including canonical Wnt, JAK/STAT, TGFb, Hh, Nuclear Receptor, Notch/Delta, and receptor tyrosine kinase (RTK) signalling (Babonis and Martindale, 2017). Small changes in this equilibrium can either result in a complete failure of development, with the generation of “monsters,” or, if increasing fitness over evolutionary time, in the diversity of organismal form and function seen today.

Downstream of these extracellular signals, mitogen activated protein kinase (MAPK) signalling is an ancient mechanism of signal transduction that has diversified significantly within the major eukaryote lineages (Xu et al., 2017; Kalapos et al., 2019), and whose dysregulation can lead to a number of diseases, including cancers (Kim and Choi, 2010). Three conventional MAPK subfamilies have been described: the extracellular signal regulated kinase (ERK), the p38 MAP kinase (p38), and the c-Jun N-terminal kinase (JNK) pathways (Krens et al., 2006; Lavoie et al., 2020). RTKs such as FGFR and EFGR mediate some of their cellular responses through the phosphorylation and activation of ERK (Shilo, 2014). ERK is often considered to be a survival factor, but it also plays important roles in cell behaviour, differentiation, and fate (Lavoie et al., 2020). JNK, on the other hand, tends to be associated with cellular migration and polarity, often as part of a non-canonical (β-catenin-independent) Wnt pathway (Gao and Chen, 2010). JNK also regulates apoptosis and the cell cycle (Pinal et al., 2019), and, similarly to p38 (Canovas and Nebreda, 2021), some stress responses.

During development, JNK proteins play a crucial role in epithelial fusion events, for instance dorsal or thorax closure in *Drosophila* (Martín-Blanco et al., 2000; Ríos-Barrera and Riesgo-Escovar, 2013), and neural tube closure in vertebrates such as mouse and chick (Krens et al., 2006; Pai et al., 2012). JNK signalling is also involved in diverse processes including gastrulation in echinoderms (Long et al., 2015); convergence extension (CE) movements in *Xenopus* (Yamanaka et al., 2002; Kim and Han, 2005); neuronal polarisation (Castro-Torres et al., 2020), spinal cord neuron development and axonal pathfinding (Schellino et al., 2019), and lateral line neuromast hair cell development and regeneration in zebrafish (Cai et al., 2016; He et al., 2016); and tail regression during metamorphosis in ascidians (Chambon et al., 2007), to only list a few. Thus, JNK is a multifunctional protein with a complex range of tissue-specific and temporally regulated activities in developing vertebrate and invertebrate embryos.

To dissect the evolution of the JNK signalling cascade in shaping morphogenetic trajectories requires the identification of both its conserved and derived functions using a comparative approach outside conventional model systems. Cephalochordates (also known as lancelets or amphioxus) represent a key phylogenetic branch for understanding the evolution of developmental mechanisms at the invertebrate to vertebrate transition (Bertrand and Escrivà, 2011). They share many fundamental developmental processes with vertebrates, including regulative development, neurulation, and somitogenesis, as well as key chordate characters such as a notochord, hollow nerve cord, post-anal tail, and the endostyle, a thyroid hormone producing organ (Bertrand and Escrivà, 2011). However, broadly speaking, lancelet genomes are much simpler, with often single orthologues of genes found in multiple copies in vertebrates, which have arisen due to the whole genome duplications in this lineage (Escrivà, 2018). They also lack the complex acquired immune systems and behaviours characteristic of many model systems (Yuan et al., 2014).

Recent research in amphioxus has provided considerable knowledge on the mechanisms underlying axial patterning, germline formation, nervous system development, regeneration, genome architecture, and gene regulation (Somorjai et al., 2012a,b; Marlétaz et al., 2018; Lin et al., 2020; Bozzo et al., 2021). Much of this has been facilitated by recent advances in “omics,” including transcriptomics, genomics and single cell, and the advancement of new methodologies including knockdown and microscopy (Escrivà, 2018). At this time, the community has achieved a good understanding of the gene repertoires of major signalling pathways, such as RTKs (D’Aniello et al., 2008), or how core signalling pathways such as Wnt (Somorjai et al., 2018; Kozmikova and Kozmik, 2020), Nodal (Li et al., 2017), and FGF (Bertrand et al., 2011), among others, govern patterning and cell fate. However, comparatively little is still known about the cellular mechanisms downstream underlying some of the key processes governing morphogenesis.

Here, we aimed to fill this gap in our understanding by studying the role of JNK signalling in regulating amphioxus development. An early study identified a single JNK gene in the genome of the Florida amphioxus, *Branchiostoma floridae* (Bertrand et al., 2009). Moreover, JNK is known to undergo Nova-mediated alternative splicing in amphioxus, similarly to vertebrates (similar direction of exon inclusion events, Irimia et al., 2011). However, nothing has so far been reported on a role for JNK signalling in morphogenesis. Based on research in other systems, we hypothesised that amphioxus JNK might regulate cellular behaviours during gastrulation and neurulation, such as epithelial fusion, neural tube closure and convergence-extension processes. Here, we show that pharmacological inhibition of JNK activity using the small molecule SP600125 causes dramatic shortening of the A/P axis and considerable cellular disruption, including loss of many anterior structures and perturbed notochord formation. We propose that an appropriate balance of JNK signalling is required to maintain cellular integrity and polarity, and that mis-regulation results in cells delaminating through changes in the actin cytoskeleton and phosphorylated ERK (pERK), culminating in Caspase-mediated cell death of depolarised cells. We also show that at least some of these phenotypes are accompanied by changes in transcriptional timing, loss, and expansion of gene expression. Specifically, we see early upregulation of *wnt11* in posterior ectoderm concomitant with reduced expression in somites and at the hinge, and an almost complete loss of *six3/6* and *wnt2* in the notochord. Taken together we provide a plausible scenario for how JNK activity orchestrates complex morphogenetic processes to govern axial elongation and differentiation to ultimately shape the cephalochordate body plan.

## Materials and Methods

### Embryo Collection and Treatments

Adult *Branchiostoma lanceolatum* were collected in Argelès-sur-Mer, France, by manual sieving of substrate as previously described (Fuentes et al., 2007). Spawning was according to previously published methods (Fuentes et al., 2007). Briefly, gravid males and females were subjected to a thermal shock for 36 h, returned to ambient temperature (19°C) and allowed to spawn naturally after sunset in individual cups containing seawater. Sperm and eggs were mixed in 0.22 μM filtered seawater to ensure consistent and timed fertilisation and maintained at 19°C for the entire developmental period.

Drug treatments and control vehicle were administered directly into petri dishes containing filtered seawater and embryos at appropriate stages, in this case right at the blastula stage (B stage; staging according to Carvalho et al., 2021) and at the end of gastrulation (G6), when the neural plate could be seen to begin flattening but while the blastopore was still open. For JNK inhibition, the cell permeable c-Jun N-terminal kinase inhibitor SP600125 (JNK Inhibitor II, Calbiochem) was used at concentrations of 2.5, 5, or 10 μM after initial pilot experiments. For Caspase inhibition, the cell-permeable, irreversible pan-Caspase Caspase inhibitor I (Z-VAD-fmk, Calbiochem) was used at 200 μM. DMSO was added at an equivalent concentration for all control experiments. Embryos were fixed in freshly prepared 4% PFA in MOPS buffer in filtered seawater overnight at 4°C, followed by several washes and storage in 70% ETOH at −20°C (WMISH), or for 2 h at room temperature (RT) followed by at least three washes and storage in 1× NaPBS at 4°C (immunohistochemistry and Phalloidin staining, respectively). For SP600125 and Z-VAD-fmk and combined inhibitor titration experiments, 50–100 embryos were used at each concentration. Definitive experiments were performed on 2–3 independent batches of embryos derived from single fertilisations (i.e., gametes from one mother and father) in four spawning seasons (2008–2011), each consisting of 500–1000 embryos each, to guarantee reproducibility. This resulted in batches of at least 200 embryos per treatment group and time point.

### Immunohistochemistry

Treated embryos and stage-matched controls were prepared for immunohistochemistry and F-Actin staining as per previous protocols (Somorjai et al., 2012a,b) unless otherwise described. Briefly, after three washes in 1× TBS (Tris Buffered Saline), embryos were permeabilised using 0.5–1% Triton in 1× TBS for 40 min at RT. After copious washing in 1× TBS supplemented with 0.1% Tween (TBST), embryos were incubated with primary antibodies overnight with rocking at 4°C. After multiple washes in 1× TBST, a blocking step was included for 2 h at RT in 5% heat inactivated sheep serum supplemented with 2% BSA prior to adding secondary antibodies. To detect Caspase-3-mediated cell death, the anti-cleaved Caspase-3 (cCasp-3) primary antibody (Asp 175; Cell Signaling Technology) was used at 50 μM. Monoclonal antibody clones DP311 and DP312 (kindly provided by Nipam Patel, University of Berkeley, CA, United States) were used at 1:30 as previously described (Somorjai et al., 2012b) and label both Pax3/7 protein products (Barton-Owen et al., 2018). Proliferation was monitored using the phospho-histone H3 (PH3) antibody at 1:500 (Millipore). α-Acetylated tubulin (Sigma) was used at 1:500 to label cilia and axons, the C2206 antibody (Sigma) to label β-catenin at the membranes (Somorjai et al., 2012a) and the phosphorylated p44/42 MAPK (ERK1/2) Thr202/Tyr204 antibody (Cell Signaling Technology) to label pERK. When used, F-Actin was visualised using Alexa Fluor 488 or Alexa Fluor 568 conjugated Phalloidin (1:200, Molecular Probes) at RT for 2–4 h or in the final overnight washes along with 1:400 Alexa Fluor secondary antibodies (Molecular Probes) at 4°C. Embryos were rinsed in 1× TBST, then washed in DAPI staining solution (1:5000 of 5 mg/ml stock) for 20–40 min at RT to visualise nuclei, followed by final washes and mounting in ProLong Gold Antifade medium for fixed cells (Thermo Fisher Scientific). All immunohistochemistry experiments were conducted at least in duplicate on 5–10 embryos per control/treatment group. A random selection of embryos was mounted on slides for imaging by confocal microscopy (3–10 embryos). Confocal images were acquired with a Leica SPII confocal microscope. Images were post-processed in Fiji ImajeJ version 2.1.0/1.53c (Schindelin et al., 2012) and Adobe Photoshop 2021.

### Whole Mount *in situ* Hybridisation

*Wnt1*, *wnt2*, *wnt3*, *wnt4*, *wnt5*, *wnt6*, *wnt7*, *wnt8*, *wnt9*, and *wnt11* probes are from Somorjai et al. (2008) and Somorjai et al. (2018); *pax3/7*, *chordin*, *brachyury2*, *neurogenin*, and *MRF1* (originally *MyoD*) are from Somorjai et al. (2008); *otx*, *foxQ2*, and *six3/6* are from Albuixech-Crespo et al. (2017); *musashi* is from Dailey et al. (2016); and *snail* is from Bertrand et al. (2011). Fragments of *dkk1/2/4*, *dkk3*, *sfrp1/2/5*, *sfrp3/4*, *fz1/2/7*, *fz4*, *fz5/8*, and *fz9/10* genes were amplified by PCR using a “touchdown” programme (Don et al., 1991) on pooled embryonic cDNA and cloned into pGEMT-Easy (Promega) or pBlueScript II KS+ (Invitrogen) vectors using standard procedures (Dailey et al., 2016). See Supplementary File 1 for sequences of probes used. WMISH was performed as previously described (Somorjai et al., 2008) after stage-dependent permeabilization with 1.125 U/ml Proteinase K (Roche). DIG-labelled (Roche) antisense probes were *in vitro* transcribed using T3, SP6, or T7 polymerase (NEB) as appropriate. The chromogenic reaction was performed in the dark for 2 h or up to several days, with daily changes of staining solution, using BMPurple (Roche) or NBT/BCIP (Roche). Embryos were mounted on slides in 80% glycerol and imaged using a Leitz DMRB microscope (Leica Microsystems) with Nomarski optics. All WMISH experiments were conducted at least in triplicate with 10–20 embryos per control/treatment group. Consistency of expression was assessed, and a representative selection was photographed (3–10 embryos). Images were post-processed in Adobe Photoshop 2021.

### Bioinformatics and Comparative Structure Modelling

*Branchiostoma floridae* and *Branchiostoma belcheri* JNK sequences were identified by tBLASTn search using human MAPK8, MAPK9, and MAPK10 protein sequences using the publicly available NCBI database.^1^ The *B. lanceolatum* gene model BL14252 was similarly identified by querying the European lancelet genome on EnsemblMetazoa using BLAST with the human sequences.^2^ However, as the gene model appeared to have an unlikely deletion, we used transcriptomic data to manually curate the sequence to generate an almost complete consensus sequence (see Supplementary Figure 1).

A comparative model of the *B. belcheri* JNK MAP kinase, residues N47 to S403, was created using Modeller 10.1 (Webb and Sali, 2016) based on the coordinates of residue D45 to N400 of human MAPK10 (1PMV) (Scapin et al., 2003). A structure-based sequence alignment of the amphioxus and human sequence was created using Clustal Omega^3^ and manually edited to ensure that secondary structural elements remained gap-free. The sequence identity was 84%. Model geometry was validated using PROCHECK (Laskowski et al., 1993). The Ramachandran plot showed that the comparative model had 94.9% of residues in the most favoured conformations and 5.1% in the additionally allowed regions. There were no residues with disallowed conformations. The superposition of 1PMV and the model gives a root mean square deviation of 0.42 Å for the backbone atoms.

## Results

### JNK Plays a Role in Invagination and Elongation in Amphioxus

In *Drosophila*, there is a single JNK orthologue, but in humans, there are three JNK genes (JNK1, 2, and 3 or MAPK 8, 9, and 10) resulting from the two whole genome duplications in the ancestor of the vertebrate lineage (Dehal and Boore, 2005). A previous study identified a single JNK orthologue in the *B. floridae* genome (Bertrand et al., 2009). However, because the increase in genomic resources available for amphioxus species has highlighted a number of unexpected lineage specific genome duplications (e.g., Barton-Owen et al., 2018), we took advantage of more recent data, including availability of the European amphioxus (*B. lanceolatum*) genome (Marlétaz et al., 2018), to validate this result. BLAST search using the human proteins confirmed the existence of a single orthologue of JNK in *B. lanceolatum*, *B. belcheri*, and *B. floridae* (Supplementary Figure 1). Sequence similarity suggests that they are highly conserved, and structurally similar to vertebrate proteins. We therefore surmised that a pharmacological approach using the small molecule inhibitor SP600125 (Bennett et al., 2001), which has affinity for all three vertebrate orthologues, would specifically target amphioxus JNK function.

**FIGURE 1:**
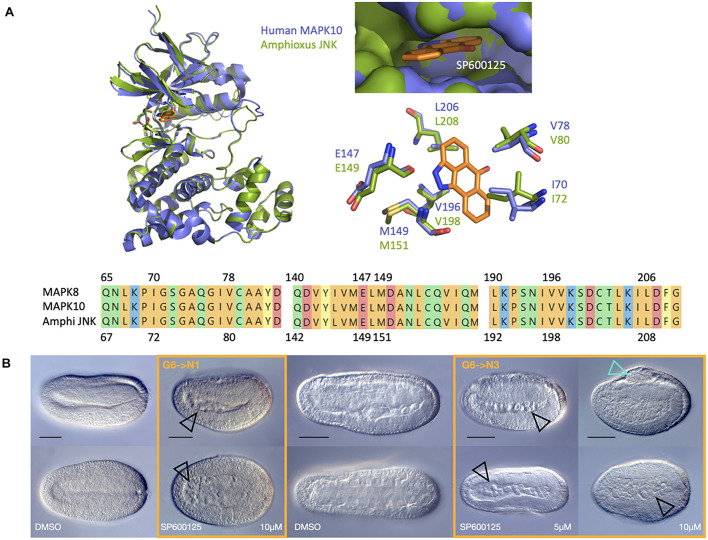
SP6000125 is a specific JNK inhibitor in amphioxus and causes axis elongation defects. **(A)** Comparative modelling of the amphioxus JNK (green) in complex with the small molecule inhibitor SP600125 (orange) using human MAPK10 as a template (purple) reveals conservation of docking sites at conserved residues (top). The alignment of human MAPK8, human MAPK10, and amphioxus JNK shows 100% identity of residues within the actives site (bottom). **(B)** SP6000125 causes dose-dependent phenotypes when amphioxus embryos are treated at the late gastrula stage. The top row shows lateral views, with anterior to the left and dorsal up. The bottom row shows dorsal views. Treatments are boxed in orange, with their stage matched DMSO controls to the left. Black outlined arrowheads highlight an accumulation of cells within the gut after only short treatments with 10 μM SP600125. Over time, all embryos exposed to the JNK inhibitor show elongation defects, and by N3, in addition to accumulation of cellular material within the archenteron (black arrowheads), treated embryos have fin-like dorsal protrusions (cyan outlined arrowhead) at 10 μM SP600125. Representative embryos are shown. DMSO control, N1 *n* = 170; 10 μM SP600125, N1 *n* = 126; 5 μM SP600125, N3 *n* = 158. Scale bars = 50 μM.

Comparative modelling of the structure of amphioxus JNK bound to SP600125 relative to known conformations of MAPK8 and MAPK10 with the inhibitor revealed that both the binding pocket and the drug’s docking sites are conserved (Figure 1A). Taken together, these data suggest that a pharmacological approach using SP600125 to inhibit JNK activity will be highly specific in amphioxus.

We initially tested several SP600125 concentrations from 1 to 20 μM, beginning at blastula or late gastrula stages. All embryos treated up to 5 μM at blastula stages (B stage) arrested during gastrulation (227/227; compare to DMSO controls 0/247; two-tailed *z* = 11.74, *P* < 0.00001), producing cap-shaped or exogastrulae (Supplementary Figure 2, and see below), but were alive based upon the normal though delayed initiation of ciliary movement. Those treated at late gastrula stages (G6) consistently developed shortened axes relative to controls (Figure 1B), with the strength of phenotype occurring in a dose-dependent manner from 2.5 to 10 μM. All embryos, regardless of treatment concentration, continued to swim in a directional spiralling fashion toward light sources (not shown). At the stronger concentrations and later stages of development, besides being short and stubby, embryos possessed a dorsal fin-like protrusion (cyan arrowhead), leading to the designation of “*orca*” phenotype. Over time, cells also apparently accumulated within the archenteron or gut cavity (black arrowheads), suggesting that JNK inhibition leads to polarity or CE defects through cellular extrusion into luminal spaces.

### Gene Expression in Anterior Endoderm and the Notochord Are Modulated by JNK Activity

Analysis at the gross morphological level suggested that, though smaller, the *orca* embryos nevertheless possessed all major tissue types, including nerve cord, notochord, and somites, albeit disorganised. Embryos allowed to develop to late neurula (N4) or pre-mouth stages (N5 or T0) further appeared to lack anterior mesodermal/endodermal structures. In order to assess more specifically which tissues were affected, we performed WMISH using a bank of 28 genes, including markers for the ectoderm and nervous system (e.g., *neurogenin*, *distalless*, and *snail*), somites and muscle (e.g., *pax3/7* and *MRF1*), the notochord (*brachyury*, *chordin*, *musashi*, and *six3/6*), anterior endoderm (*otx* and *six3/6*) as well as markers for embryonic polarity (e.g., *nodal*, *foxQ2*). We also included a large number of Wnt signalling components, from ligands (*wnt1–9* and *wnt11*) to receptors (*fz1/2/7*, *fz4*, *fz5/8*, and *fz 9/10*) and antagonists and co-receptors (*sfrp4*, *sfrp1/2/5*, *dkk1/2/4*, and *dkk3*) for two reasons. First, the Wnt pathway defines a wide range of cell and tissue types in amphioxus (Somorjai et al., 2018). Second, there is some evidence, mostly in vertebrates, for cross-talk between the JNK and other Wnt pathways, both at protein and transcriptional levels (Hardy et al., 2008; Lee et al., 2009; Seo et al., 2010; Mazzotta et al., 2016). JNK has even been shown to phosphorylate β-catenin to regulate adherens junctions and cellular adhesion (Lee et al., 2009; Zeke et al., 2016). We therefore hypothesised that (1) convergence-extension defects might be uncovered through the analysis of markers for neural and notochord tissues, specifically, although morphogenesis of other tissues may also be affected; and (2) we might detect transcriptional and protein-level responses of the Wnt pathway, including feedback loops, to treatment with SP600125.

We focused primarily for this analysis on embryos treated with 10 μM and allowed to progress to mid-neurula stages (N2 or N3) as this produced a good balance between strength of phenotype and good progression of development and differentiation. Gene expression assessed in embryos treated at the blastula stages was either too difficult to compare with early neurula stage controls due to the early arrest in gastrulation, or closely matched that seen in wild type gastrulae (Supplementary Figure 2). We did not assess these early phenotypes further here.

Taking into consideration the fact that embryos were generally more compact, and therefore some compression or distortion of expression domains is to be expected, our candidate approach revealed four classes of result. In some cases, a particular marker may show no expression change for one anatomical structure or germ layer, and yet differ significantly between controls and treated embryos for another, and thus may find itself within more than one group.

#### Genes Showing Broad Maintenance of Normal Gene Expression

**FIGURE 2:**
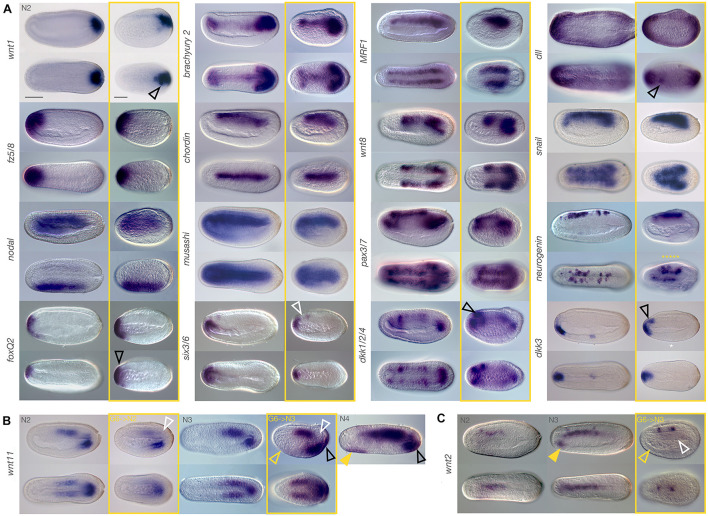
Marker gene expression after JNK inhibition at late gastrula stages is modulated. Embryos were treated with 10 μM SP600125 and allowed to develop to N2, N3, or N4 stages (boxed in yellow). Stage matched controls are on the left. **(A)** Treated embryos generally show similar expression patterns to the controls. Exceptions include *dkk1/2/4*, *dkk3*, *dll*, *foxQ2*, and *wnt1* which show slightly expanded or ectopic expression (black outlined arrowheads), and *six3/6*, which appears to lack expression in the anterior notochord (white outlined arrowhead) despite comparable neural expression. Irregular expression of *neurogenin* in neurons along either side of the midline (yellow asterisks) is also apparent. **(B)** After treatment with the JNK inhibitor, *wnt11* expression is reduced relative to control N2 embryos (white arrowhead). By N3, chordoneural hinge expression is still absent (white outlined arrowhead), and there is no ventral endoderm expression (yellow outlined arrowhead) similarly to controls. However, ectopic expression within posterior ectoderm of the tailfin is observed (black outlined arrowhead). This is more similar to N4 embryos (black outlined arrowhead) than to the stage matched N3 controls, but does not fit with anterior ventral endoderm expression in these later stage embryos (filled yellow arrowhead). **(C)** Expression of *wnt2* is also dysregulated by JNK inhibition in the notochord (white outlined arrowhead) and in anterior ventral endoderm (yellow outlined arrowhead), relative to stage matched N3 controls, which also have expression in the notochord (compare to N2 embros). This occurs in spite of normal neural expression, and cannot be explained by a systemic delay of development. Representative embryos are shown. Scale bars = 50 μM.

Many genes showed no major change in gene expression. Generally, markers for embryonic polarity fall within this category. This includes *wnt1*, expressed at the most posterior of the embryo in the tailbud; *fz5/8*, expressed in anterior ectoderm and endoderm; and *nodal*, expressed asymmetrically on the left side in both treated and control embryos (Figure 2A). *Brachyury2*, *chordin*, and *musashi* appear to be expressed normally within the notochord domains, although the domain itself may be somewhat mediolaterally expanded in line with the shortened axis. *Pax3/7*, *wnt8*, and *MRF1* are expressed in somites. Neural domains are broadly congruent, as assessed by *chordin*, *pax3/7*, and *neurogenin*, although the latter shows clear disorganisation of neural positioning (Figure 2A, yellow asterisks), possibly reflecting epithelial fusion defects caused by JNK inhibition. *Dll* is expressed most strongly in anterior and posterior ectoderm, with some expression along neural folds, in both control and treated embryos. Other genes exhibiting no discernible change in expression domain or inconclusive results include *fz1/2/7*, *fz4*, *fz9/10*, *wnt7*, *wnt6*, *wnt3*, *wnt4*, and *sfrp1/2/5*, at least when assessed by N2/N3 (Supplementary Figure 3 and not shown).

#### Subtle Changes in Extent of Expression or Patterning Within Specific Domains

We also found that some expression domains either extended further than expected or were reduced relative to the controls. For instance, anterior ventral endodermal expression of *dkk3* was expanded dorsally in treated embryos to include cells in close apposition to, or possibly within the anterior neural plate/cerebral vesicle (Figure 2A, black outlined arrowhead). Conversely, *six3/6* expression was maintained in the cerebral vesicle and anterior-most endoderm but was absent in presumptive anterior notochord (as per Albuixech-Crespo et al., 2017) in treated embryos (white outlined arrowhead). Likewise, the dorsal-ventral extent of expression of *foxQ2* appeared similar in control and treated embryos, but in fact extended further in lateral domains when viewed dorsally (black arrowhead). Anterior expression of *snail* was also reduced in treated embryos relative to controls. Finally, *dkk1/2/4* appeared strongly expressed specifically in the anterior-most pair of somites as well as the anterior neural plate/cerebral vesicle (black outlined arrowhead) in treated embryos compared to controls.

#### Ectopic Expression

Generally, true ectopic expression was rare and isolated to only few cells and may reflect tissue disorganisation resulting from JNK inhibition. In treated embryos, we found cases of clusters of cells outside their normal domain expressing *dll* and *wnt1* (Figure 2A, black outlined arrowheads) in ectodermal neural fold and tailbud cells, respectively. More convincingly, in treated embryos allowed to develop to the N3 stage, we also saw strong ectopic expression of *wnt11* in the posterior ectoderm of the tail fin (black outlined arrowhead). This domain of expression appears much later in wild type embryos, after N4. We also found that treatment with SP600125 resulted in the cerebral vesicle and anterior endoderm expression domains of *otx* to merge, reflecting either ectopic expression or a loss of separation between the two tissue types (Supplementary Figure 3, black outlined arrowhead).

#### Loss of Expression

Very few genes demonstrated a complete loss of expression, with three exceptions. *Dkk3* shows expression within a cluster of ventral endodermal cells in control embryos, a pattern consistently absent in treated embryos (Figure 2A, white asterisk). However, we found that this phenotype had variable penetrance even in wild type embryos (not shown), possibly reflecting slight delays in development, and should be considered with caution. *Wnt11* expression appears highly reduced in the last enterocoelic somites after treatment at N2, despite strong posterior ventral endoderm expression, and appears absent in the presumptive notochord cells within the hinge region of the tailbud (Figure 2B, white outlined arrowhead). Allowing embryos to develop further until at least N3 results in expression patterns resembling those of much later embryos (wild type N4 stage, rightmost panel), with the exception of continued absence of hinge expression (white outlined arrowhead), as well as complete absence of anterior ventral endoderm expression (yellow outlined arrowhead), which is characteristic of matched N3 controls but not of later N4 stages (filled yellow arrowhead). This suggests a loss of expression or cells within the anterior endodermal *wnt11* domain. Most notable in this category is expression of *wnt2* in treated embryos at N3, which have strong expression in at least two (sometimes three) clusters within the nerve cord, similar to stage-matched control embryos (Figure 2C). In contrast, they entirely lack notochordal *wnt2* expression (white outlined arrowhead), or anterior endodermal expression (outlined yellow arrowheads) relative to the control (filled yellow arrowhead). Although also lacking endodermal expression, the earlier N2 stage wild type embryos have consistent notochordal expression. Extension of the chromogenic reaction only strengthened the nerve expression, with background staining in other tissues. This result is particularly striking when compared to the strong expression of notochord markers, in particular *brachyury2*.

Taken together, and as expected if one of the functions of JNK is to mediate cellular behaviour, our results indicate that transcriptional changes in many key patterning and cell type specification genes are relatively unaffected by JNK inhibition both during gastrulation and neurulation. However, we did see several marked changes, particularly of gene expression domains in the anterior endoderm, as well as shifts in relative contribution to different tissues of genes such as *wnt11*, and most notably, loss of *six3/6* and *wnt2* in the notochord.

### Inhibition of JNK Causes Significant Cellular Disruption

Given the *orca* phenotype consists of a shortening of the axis, some tissue disorganisation and a number of changes in gene expression domains, but that broadly speaking cell and tissue types appear to be maintained, we wanted to assess how JNK inhibition might be mediating these effects at the cellular level. We therefore utilised Phalloidin staining to assess any changes in F-Actin as a readout for modulation of the cytoskeleton and cellular behaviours, as many cytoskeletal proteins are substrates of JNK, including Actin-binding proteins and β-catenin (Lee et al., 2009; Zeke et al., 2016). In combination with other antibodies, including against β-catenin, Pax3/7, and acetylated-tubulin, we identified several tissue-specific changes that might contribute to the effects of JNK inhibition on morphogenesis in amphioxus embryos.

#### Disorganisation of the CNS

**FIGURE 3:**
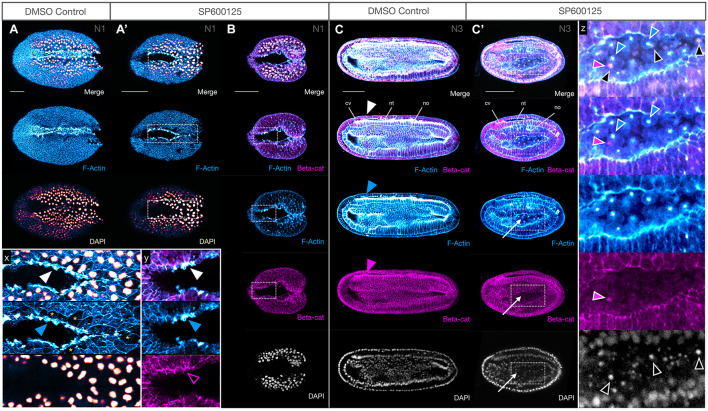
JNK inhibition produces no major defects in epidermal fusion or neural tube closure. **(A,A′)** Phalloidin staining of F-Actin (blue) in apical membranes shows epidermal fusion in SP600125-treated embryos proceeds similarly, but more slowly, in treated embryos **(A′)** relative to DMSO controls **(A)** at N1 stage. Inset x clearly shows an accumulation of F-Actin in the leading edge (white arrowheads) of fusing epidermal sheets along the midline. Cells with such protrusions experiencing tension after having just fused, or in the process of fusion, appear to be depleted in cytoplasmic F-Actin (yellow asterisks). Dorsal views are shown. **(B)** Immunohistochemistry of β-catenin (beta-cat, magenta) shows that the accumulation of F-Actin in leading edge cells is not accompanied by concomitant accumulation of β-catenin (inset y, arrowheads). Dorsal views shown. **(C,C′)** By N3, the neural folds have fused into a closed neural tube in both controls **(C)** and SP600125-treated embryos **(C′**, arrowheads) despite obvious morphogenesis defects. Cells accumulating within the archenteron contain central aggregates of F-Actin (white arrows) and pycnotic nuclei (DAPI) (inset z, coloured arrowheads show non-correspondence of nuclear fragments and F-Actin aggregates, and general absence of β-catenin except as infrequent and generally microscopic puncta). Representative embryos are shown. Lateral views shown. In all cases, anterior is to the left. cv, cerebral vesicle; nt, neural tube; no, notochord. Scale bars = 50 μM.

**FIGURE 4:**
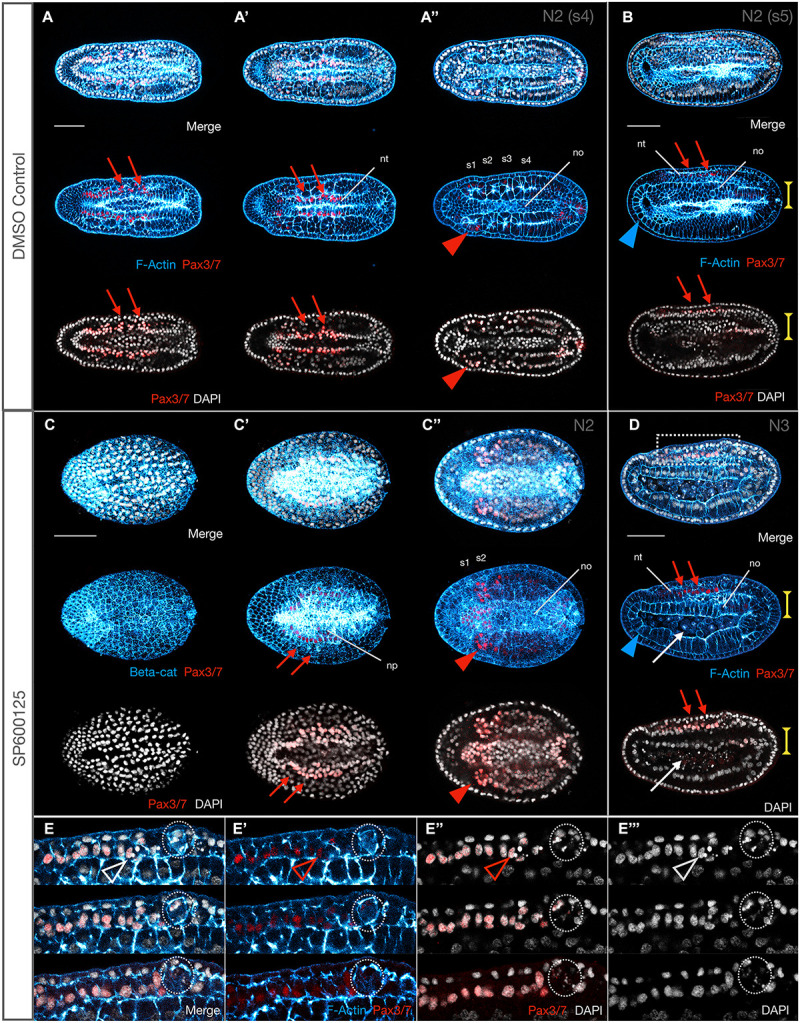
Some Pax3/7 positive neurons are extruded and die, and somite formation is delayed after JNK inhibition. **(A,A′,A″)** Pax3/7 labels neurons along the midline (red arrows). By N2 (four somite pairs), the neural tube is effectively closed. Somites are also Pax3/7^+^, particularly S1 (red arrowhead). All images are the same embryo in different focal planes (**A** most dorsal, **A″** most ventral). **(B)** Lateral view of a slightly more developed (five somite pairs) N2 stage embryo showing the Pax3/7^+^ neurons of the neural tube (red arrowheads). The anterior endoderm is comprised of a monolayer of large cells (blue arrowhead). The central notochord is 3–4 cell layers thick (vertical yellow extent lines) and similarly wide **(A″)**. **(C,C′,C″)** Stage matched SP600125-treated embryos still have neural plates, with irregular Pax3/7 expression along the edges (red arrows). Only two somite pairs can be resolved, which express Pax3/7 (red arrowhead) and the notochord is 5–6 cells wide. **(D)** By the time controls reach N3, treated embryos have caught up with neural tube closure and Pax3/7^+^ neurons are expressed along the midline (red arrowheads, compare with N2 control embryo in **B**). However, anterior endoderm is reduced (blue arrowheads) and the notochord is a monolayer of columnar cells (vertical yellow extent lines). Cells accumulating in the archenteron/forming gut are not Pax3/7^+^ and therefore unlikely to be of neural origin. **(E,E′,E″,E″′)** Insets from embryo in **D**. Each panel consists of three confocal planes, with top being the most lateral and bottom the most medial. Some Pax3/7^+^ neuronal cells are pycnotic, and therefore likely dying (outlined arrowheads), and several aggregate into extrusion masses (dotted outline) between the neural tube and the overlying epidermis. In all cases, anterior is to the left. Representative embryos are shown. Panels **(A,A′,A″)** and **(C,C′,C″)** are dorsal views; **(B)**, **(D)**, and **(E,E′,E″,E″′)** are lateral views. Pax3/7 immunoreactivity is in red, F-Actin or β-catenin (beta-cat) labelled membranes are in blue, and DAPI stains nuclei in grey. nt, neural tube; no, notochord; s1, s2, etc., numbered somites. Scale bars = 50 μM.

One of the most striking observations of the gene expression analysis was the apparent asymmetry of neuron alignment at N3 stages seen with *neurogenin* staining. We hypothesised that defects in early stages of neurulation, either of fusion of the epithelial sheets or of neural folds, might account for this patterning defect. At N1, when neurulation has begun with fusion of the epithelial cells along the dorsal midline, we saw no major differences in F-Actin between treated and control animals. In both, F-Actin accumulates at the leading edge of the zippering epithelial sheet (Figures 3A,A′), and by N3 the epidermis overlying the neural plate/tube is continuous (Figures 4A,A′,C,C′).

We also analysed the distribution of β-catenin, the nuclear effector of canonical Wnt signalling and a key component of the actin cytoskeleton at adherens junctions and membranes linking F-Actin to E-Cadherin. We found no obvious changes in accumulation of β-catenin at the adherens junctions or the membranes at early or later stages (Figures 3B,C,C′), and neurulation appears to reach completion. We did, however, see that the cells accumulating within the gut cavity of SP600125-treated embryos show central aggregation of F-Actin, which was not matched by changes in β-catenin expression (Figure 3C′, inset z) and appear to be dying as assessed by the presence of pycnotic nuclei.

Next, we assessed the organisation of the neuronal cells along the neural folds using DP311 and DP312 clones, which have previously been shown to label Pax3/7 (Somorjai et al., 2012b). In control N2 embryos, we found expression of Pax3/7 in two stripes along the dorsal midline, corresponding to neurons within the neural folds (Figures 4A,A′,A″,B). JNK inhibition results in a delay of neural plate folding, but Pax3/7 is expressed at the edges as expected for an earlier stage of development (Figure 4C′, red arrows), and by N3 expression is broadly as expected along fused neural folds (Figure 4D, red arrows). However, upon closer inspection, in addition to general disorganisation medio-laterally along the folds (also as seen with WMISH) the neurons are disorganised within the apicobasal axis, several contain pycnotic nuclei (Figures 4E,E′,E″,E″′), and groups of dying cells appear to be aggregating under the overlying epidermis (Figures 4E,E′,E″,E″′, dotted circles).

**FIGURE 5:**
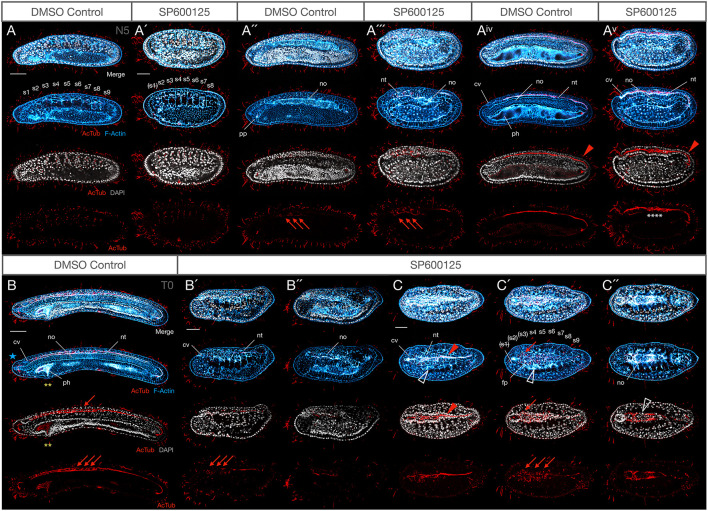
Weak JNK inhibition allows embryogenesis to proceed to N5 and T0 stages, including axonogenesis, but mesodermal structures are still affected. **(A–A^*v*^)** N5 stage control and 2.5 μM SP600125-treated embryos at equivalent confocal planes. At N5, DMSO controls possess nine somite pairs **(A)**, a clear ciliated preoral pit **(A″)**, and the central canal of the nerve cord is populated by an axonal tract extending from the neuropore of the cerebral vesicle to the neurenteric canal (**A^*iv*^**, red arrowhead). Treated embryos have no more than eight somites, and the first is degrading as shown by pycnotic nuclei [**A′**, indicated by (s1)]. There is no evidence of a preoral pit **(A″′)**, but peripheral axon tracts envelop neurons (**A″′**, red arrowheads) and axons extend through the neural tube **(A^*v*^)** similarly to controls. The notochord is highly disorganised and no pharynx proper develops. Some branching defects are evident within the central axonal tract (white asterisks). **(B–C″**) T0 stage control and 2.5 μM SP600125-treated embryos. The T0 control has more developed anterior structures, including anterior extension of the notochord past the cerebral vesicle (**B**, blue star), and cellular reorganisation of the pharynx presaging perforation (yellow asterisks). In treated embryos, the anterior endoderm remains simplified and filled with pycnotic cells **(B′,B″)** there is no extension of the notochord past the cerebral vesicle, and anterior somites continue to degenerate **(C–C″)**. Nevertheless, muscle fibres differentiate (**C,C′**, white arrowheads) and axons project contralaterally in the anterior neural tube dorsal to a pycnotic and disorganised, albeit partially differentiated, notochord (**C″**, white arrowhead). In all cases, anterior is to the left. Panels **(A–A^*v*^)** and **(B–B″)** are lateral views; **(C–C″)** are dorsal views. **(A,A″,A^*iv*^)**, **(A′,A″′,A^*v*^)**, **(B′,B″)**, and **(C,C′,C″)** are different confocal planes of the same embryos. Representative embryos are shown. Acetylated-tubulin immunoreactivity is in red, F-Actin labelled membranes are in blue, and DAPI stains nuclei in grey. cv, cerebral vesicle; fp, floor plate; nt, neural tube; no, notochord; s1, s2, etc., numbered somites; ph, pharynx; pp, preoral pit. Scale bars = 50 μM.

Finally, we used anti-acetylated-tubulin, which labels cilia and axons, to further analyse later effects on nervous system development. In other models, inhibiting JNK signalling can cause pathfinding and arborization defects (Schellino et al., 2019). For these experiments, embryos were treated at 2.5 μM, the lowest dose of SP600125, so that they could progress past N4 to N5 and T0. In both N5 controls and treated embryos, axons can be seen throughout the medial neural canal, extending from the anterior neuropore of the cerebral vesicle all the way through the neurenteric canal connecting the neural tube to the gut (Figures 5A^*iv*^,A^*v*^, red arrowhead). More lateral confocal sections further show a population of neurons on either side of the neural folds labelled with acetylated tubulin (Figures 5A″,A″′, red arrows). All structures are similarly labelled in T0 control and treated embryos (Figures 5B,B′,B″); this is particularly evident in dorsal views (Figures 5C,C′,C″). The most basal/ventral section taken under the floorplate and immediately above the notochord shows axons crossing the midline to the contralateral side, just posterior to the cerebral vesicle, despite cellular disorganisation and patterning defects (Figure 5C″). However, the branching architecture appears “messier” and axon tracts are more spread out in all treated embryos (see acetylated tubulin staining in Figures 5A^*v*^,C). We therefore cannot rule out more dramatic effects caused by more potent JNK inhibition than what we applied here.

#### Delayed and Disorganised Somite Formation and Loss of Somite Derivatives

Expression of anterior and posterior somite markers, as well as *MRF1*, an axial muscle-specific marker, suggested that specification and differentiation proceeded at least grossly normally. However, the absence of elongation might indicate delays in somite formation, or other structural changes. F-Actin accumulation at membranes not only allows identification of somite boundaries, but it also labels the differentiated muscle fibres of the myomeres starting in late neurula stages (N4). We found that formation of the first somites by enterocoely appears to proceed broadly normally at first, although it is delayed, and the effects are concentration-dependent. For instance, by N2 stages, control embryos possess 5–6 somite pairs (median 5, *n* = 7). In contrast, 10 μM SP600125-treated embryos possess 3–4 (median 3.5, *n* = 5), revealing a significant delay in somitogenesis (Mann–Whitney *U* = 0, *P* < 0.01 two tailed). That somite specification is normal is reflected by expression of Pax3/7 in the mesoderm of the first forming somites as one would expect (Compare Figures 4A″,C″). Over time, somites become more and more compressed in the A-P and D-V axes, even at the lowest concentrations of 2.5 μM SP600125 (Figures 5A,A′,B′,C′; also Figures 9C,D). Interestingly, under these conditions no treated embryos allowed to progress to the N5 stage (9–10 pairs of somites, median 9.5, *n* = 8), once somite formation progresses *via* schizocoelic budding from the tailbud, ever possessed more than 8–9 pairs of somites (median 8, *n* = 8), even accounting for evidence that the anterior-most somite(s) might be deteriorating and dying, as evidenced by pycnotic nuclei (Figures 5A′,C′; also Figures 9C,D; Mann–Whitney *U* = 0, *P* < 0.01 two tailed). In contrast, stage matched controls possess more than 11 pairs and have passed tailbud stage T0. However, although somites are disorganised in embryos treated with SP600125, they do nevertheless appear to differentiate, as the dorsal regions contain evidence of F-Actin-rich lateral muscle fibres extending between myosepta (Figures 5C,C′; open white arrowheads).

Last, our assessment reveals that JNK inhibition results in the absence of formation of the preoral pit, a monociliated organ with possible homology to the vertebrate adenohypophysis (Candiani et al., 2008). During amphioxus development, it has been argued that the ventral portions of the first somite pair split off to give rise to the left and right diverticula; the left diverticulum becomes the preoral pit (or Hatschek’s fossa) (Stach, 1996). Even accounting for a delay in development caused by pharmacological treatment, and whatever its specific origins (ventral extension of the first somite or direct pinching off from enterocoelic mesoderm), we find no evidence for the formation of a preoral pit in any of our SP600125-treated embryos (Figure 5). We also find no evidence of its formation and then subsequent degradation, as at no time is there a ciliated structure in this location.

Taken together, our results indicate that JNK inhibition causes a delay in the rate of enterocoelic somite formation but not of their maturation, with eventual degradation of anterior somite pairs, and an absence of preoral pit formation.

#### Notochord Convergence and Extension Disruption Is Accompanied by Cellular Extrusion

**FIGURE 6:**
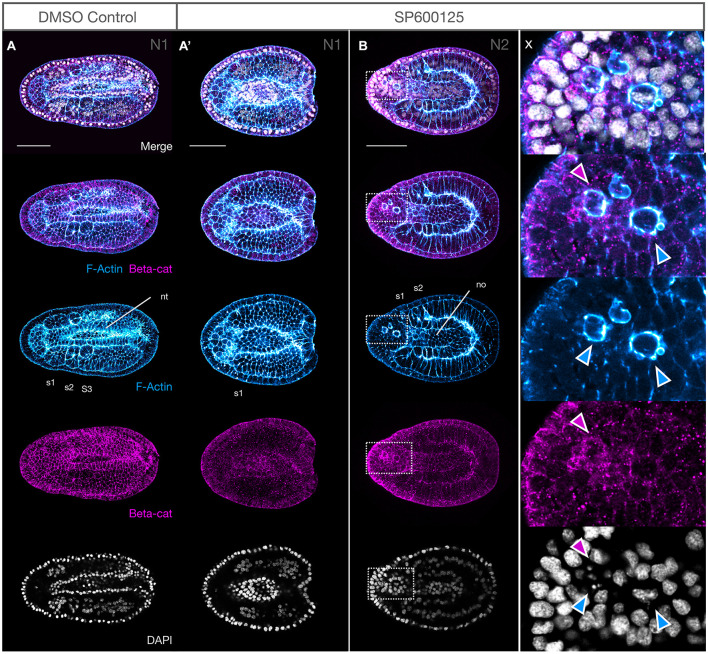
Notochord cells round up, accumulate F-Actin and β-catenin at the membranes and undergo nuclear breakdown after JNK inhibition. **(A,A′)** At N1, the membranes of DMSO controls contain F-Actin and β-catenin, with three pairs of somites clearly outlined. The neural tube is almost entirely closed, and notochord cells can be seen anteriorly and below **(A)**. SP600125-treated embryos are delayed, with only the first somite demarcated. β-Catenin is patchily distributed but outlines notochord cells, particularly their irregular apical membranes anteriorly **(A′)**. **(B)** By N2, some notochord cells have rounded up, with strong F-Actin staining in the membranes and accumulation of β-catenin (magenta arrowheads in inset x). These cells have numerous blebs (blue arrowheads) and DAPI-stained nuclei are irregular or pycnotic (inset x). All embryos are orientated anterior to the left, with dorsal views shown. Representative embryos are shown. F-Actin and β-catenin labelled membranes are in blue and magenta, respectively, and DAPI stains nuclei in grey. nt, neural tube; no, notochord; s1, s2, etc., numbered somites. Scale bars = 50 μM.

The observation that somite formation and differentiation is delayed led us to query the mechanism leading to the notochord elongation defect seen in SP600125-treated embryos. While shorter than N3 stage controls, the notochords of treated embryos appeared multi-layered, although some cells showed particularly strong F-Actin accumulation in membranes (Figure 3C′). We therefore looked at earlier stages of development to determine whether JNK inhibition caused any changes in cellular behaviour in the notochords of treated embryos. Indeed, along with a delay in somitogenesis, notochords of treated embryos are both broader (Figure 4C″) and dorso-ventrally shallower (Figure 4D, yellow extent lines) than their N2 controls (compare with A″ and B). In other words, the notochord fails to extend in the antero-posterior and dorso-ventral axes in a classic convergence-extension phenotype, remaining as a monolayer even in N3 stage embryos. Careful analysis of embryos at N1 (Figure 6A,A′) and N2 stages (Figure 6B and inset x) indicates that some cells begin to show irregular contours at N1, followed by rounding up and blebbing. Membranes accumulate F-Actin as well as β-catenin (Figure 6B and inset x), and nuclei begin to fragment (inset x, note DAPI staining in rounding cells).

Even with weaker treatments with SP600125 (2.5 μM) and allowing embryos to develop to N5 or T0 stages, the notochords remain disorganised relative to those of controls, lacking the classic “stacked coin” morphology of their matched controls (Figures 5A^*iv*^,B vs. A^*v*^,B″). By these late stages, many notochord cells are pycnotic (Figure 5C″, grey outlined arrowhead) and there is no anterior notochord extension past the cerebral vesicle as seen in T0 controls (Figure 5B, blue star).

Taken together our results suggests that a delay in differentiation is not sufficient to account for the CE defects seen in embryos after JNK inhibition. Cellular extrusion starting early on also accounts for the shorter, disorganised notochords seen after SP600125 treatment.

### Changes in Proliferation Are Not the Prime Mediator of Size

JNK is known to play an important role in cellular survival, as well as regulation of the cell cycle (Pinal et al., 2019; La Marca and Richardson, 2020). We hypothesised that growth and patterning defects might be caused by dysregulation of the balance between cell death and proliferation, either through a broad reduction in proliferation or through changes in patterns of cell division in different tissues. We therefore compared proliferation between treated and control embryos using an antibody against PH3, a marker commonly used to detect late G2 and mitosis phases of the cell cycle. Since embryos at higher levels of drug concentration (10 μM), or those allowed to develop for a long period even at lower concentrations (2.5 μM) showed extreme changes in morphology and reduction in size, complicating interpretation, we first assessed proliferation in N3 stage control and 5 μM – treated embryos, an interval permitting sufficient time for the drug to act without causing major cellular shedding into the archenteron/gut cavity.

**FIGURE 7:**
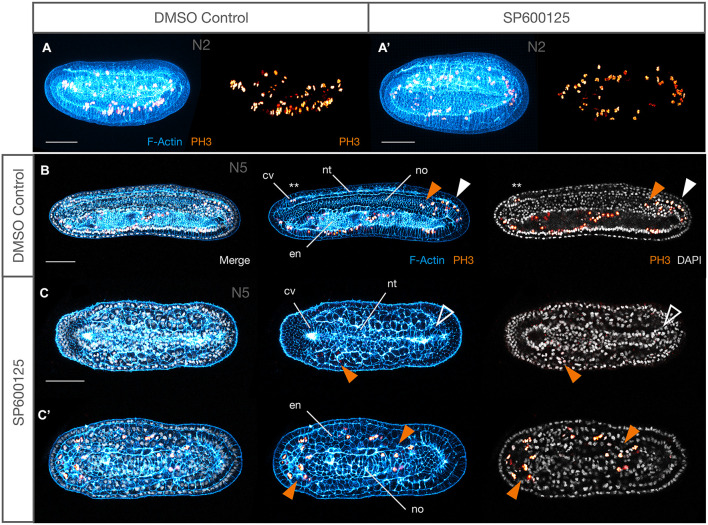
Proliferation is maintained after JNK inhibition. **(A,A′)** Maximum projections of all confocal planes in control and SP600125-treated embryos stained for F-Actin (cyan) and phospho-histone H3 (PH3, gold) at the N2 stage showing broad distribution of mitotic cells. **(B)** Control N5 and **(C,C′)** stage matched treated embryos showing patterns of proliferation in different tissue types. In controls **(B)**, proliferation is found throughout the endoderm, but otherwise mostly localised to the posterior neural tube (white arrowhead) and notochord (orange arrowhead), and rarely in somites (not shown) or the cerebral vesicle (asterisks). In spite of dramatic size reduction in treated embryos **(C,C′)**, endoderm contains many mitotic cells as does the posterior notochord, and unusual examples of PH3^+^ somatic cells can be seen (orange arrowheads). No neural cells divide here, even in the posterior neural tube (white outlined arrowhead). Representative embryos are shown. In all cases anterior is to the left. Panels **(A,B)** are lateral views, **(C′,C)** are dorsal views of the same embryo at different confocal planes. cv, cerebral vesicle; en, endoderm; nt, neural tube; no, notochord. Scale bars = 50 μM.

Although total cell number is difficult to accurately quantify in entire amphioxus embryos, maximum projection of all sections containing PH3-positive cells shows a broadly similar distribution and density of proliferating cells (representative embryos in Figures 7A,A′). By N5, there is a clear difference in size of the treated embryos, which consist of absolutely many fewer cells. Nevertheless, comparison of proliferation patterns again does not indicate any clear differences. In the control, PH3^+^ cells are found in the cerebral vesicle and somites, though rarely, as well as throughout the endoderm, and are particularly prevalent in the tailbud region, including posterior nerve cord and notochord (Figure 7B). There is little to no proliferation in the rest of the nerve cord or notochord. This, and the inter-individual variability in proliferation patterns, is consistent with what has been observed in other studies (Holland and Holland, 2006; Carvalho et al., 2021). Similarly, mitotic indices are rare in the nerve cord, along the notochord axis and in the cerebral vesicle in embryos treated with SP600125. However, considerable proliferation can be seen in both anterior and lateral endoderm, as well as in the posterior of the notochord (Figures 7C,C′). Taken together, our results suggest that proliferation does continue throughout the tissues, even after a long exposure to the JNK inhibitor, but may not be sufficient to compensate for cellular losses. However, deeper analysis of cell cycle dynamics in large sample sizes will be required to confirm these initial observations.

### Inhibiting JNK Signalling Causes Caspase-3-Dependent Apoptosis

**FIGURE 8:**
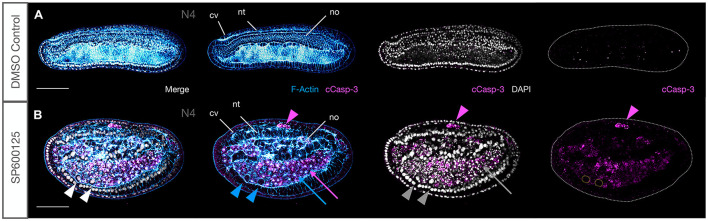
JNK inhibition induces Caspase-3-mediated apoptosis. **(A)** Under ordinary control conditions, there is no Caspase-3-mediated apoptosis in N4 embryos. **(B)** JNK inhibition with SP600125 causes dramatic accumulation of cleaved Caspase-3 in cells that have been extruded between the neural tube and epidermis (magenta arrowhead), around the notochord and within the accumulated cells of the archenteron where pycnotic nuclei (grey arrow) and F-Actin aggregates (blue arrow) are observed. Rounded cells that have delaminated with strong membrane staining and no F-Actin aggregates have intact nuclei and are cCasp-3^–^. Note how ventral cells highlighted by white, blue, and grey arrowheads lack cCasp-3 staining in single magenta channel (cells outlined with yellow dotted lines). Representative embryos are shown. Phalloidin staining of F-Actin is in blue and cleaved Caspase-3 (cCasp-3) immunoreactivity is in magenta, while DAPI stained nuclei are in grey. Embryos are orientated with anterior to the left and dorsal up. cv, cerebral vesicle; nt, neural tube; no, notochord. Scale bars = 50 μM.

**FIGURE 9:**
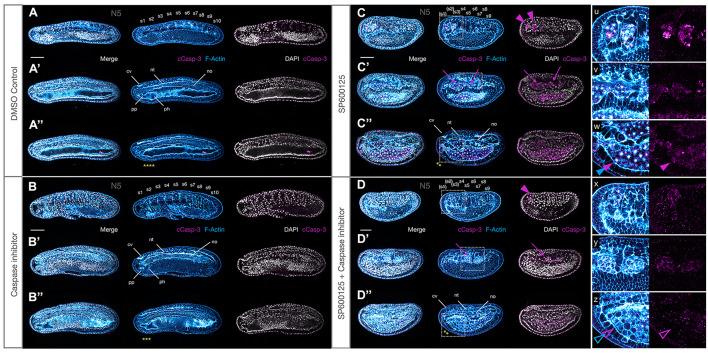
A pan-Caspase inhibitor only partially rescues weak JNK inhibition phenotypes. **(A,A′,A″)** N5 stage DMSO control with 10 somite pairs **(A)**, a preoral pit **(A′)**, and expanding pharyngeal region (**A″**, yellow asterisks). **(B,B′,B″)** Stage matched embryo treated with the pan-Caspase inhibitor Z-VAD-fmk at high concentration (200 μM). Embryos are slightly shorter but otherwise equivalent to the controls, with all differentiated structures present. **(C,C′,C″)** Weak JNK inhibition with 2.5 μM SP600125 shows dramatic accumulation of cleaved Caspase-3 in dying cells, including degenerating anterior somites (**C**, s1–s3, magenta arrowheads; inset u), cells extruded around the notochord (**C′**, magenta arrows, inset v), and within the archenteron (**C′**, inset w). No real pharyngeal region has differentiated (yellow asterisks). **(D,D′,D″)** Embryos co-treated with SP600125 and Z-VAD-fmk are grossly similar to embryos treated with SP600125 alone. However, cleaved Caspase-3 immunoreactivity is significantly reduced both quantitatively and qualitatively. The notochord, although still delayed, has a more regular cellular arrangement (compare **C″,D″**), and cellular protrusions (insets x and y) contain rounded cells, but fewer central F-Actin aggregates. Rounded cells in the anterior of the archenteron have a similar “healthier” phenotype (compare w and z, filled and outlined blue and magenta arrowheads), with evidence that at least some may originate from within the anterior ventral endoderm layer. Phalloidin staining of F-Actin is in blue and cleaved Caspase-3 (cCasp-3) immunoreactivity is in magenta, while DAPI stained nuclei are in grey. Embryos are orientated with anterior to the left and dorsal up. Each panel series shows one representative embryo at different confocal planes. cv, cerebral vesicle; nt, neural tube; no, notochord; s1, s2, etc., somite pairs. Scale bars = 50 μM.

The observation that the large number of cells within the archenteron or between tissues often contained pycnotic nuclei suggested that JNK-mediated loss of polarity might be associated with the activation of cell death pathways. Caspases are typically activated during apoptosis and can be detected using an antibody against cleaved Caspase-3. We found a strong and persistent expression of anti-cCasp-3 in all embryos treated with the JNK inhibitor SP600125 at all concentrations used and at every stage tested (Figure 8B). In contrast, immunoreactivity was never detected in tissues of control animals (Figure 8A and Supplementary Figure 4), with the exception of an occasional shed cell within the gut lumen in N4-T0 stages (e.g., Figures 9A′,A″). Unexpectedly, even in treated embryos, cells within tissues very rarely expressed the apoptosis marker and it was mostly confined to cells within the extracellular spaces within the archenteron/gut, in the extruded “dorsal fin” cells, or within the open space between the nerve cord and overlying epidermis (Figure 8B, magenta arrows and arrowhead). In early neurula stages, cells from the gut lumen may have squeezed through the neurenteric canal while still open. However, examples of isolated cases of cCasp-3^+^ pycnotic nuclei in CNS and chordamesoderm cells undergoing extrusion in strong early treated embryos suggest that there may be several origins of delaminated cells. We never saw an example of cCasp-3 expression in gut cells proper, but rounding of anterior ventral endoderm cells, particularly in strong early treated embryos, and accumulation of such cells within the anterior gut suggest that JNK inhibition might also lead to delamination of these cells and their subsequent death (Figure 8B, blue arrows and arrowhead). Taken together, these data suggest that Caspase-3-mediated apoptosis may at least in part be a secondary effect of loss of polarity and positional identity.

### Pharmacological Inhibition of Caspase Activity Partially Rescues the *orca* Phenotype

Loss of JNK activity might either directly activate Caspase-dependent apoptosis, or alternatively, indirectly cause apoptosis in cells that lose polarity and leave their assigned tissue. In order to attempt to distinguish between the two hypotheses, we performed a series of experiments to determine whether or not we could “rescue” the *orca* phenotype by co-treating embryos with SP600125 and the pan-Caspase inhibitor Z-VAD-fmk and comparing with individual treatments. Z-VAD-fmk is a pan-Caspase inhibitor that has been shown to block amphioxus Caspase-3/7 activity *in vitro* (Bayascas et al., 2002), but the phenotypes were never assessed *in vivo*. Because of the strength of the phenotype and the need to increase the developmental time window to assess phenotypic effects, we combined a strong Z-VAD-fmk treatment (200 μM) with the lowest 2.5 μM SP6000125 treatment condition, allowing embryos to develop with clearly discernible morphology to N4 or even T0 stages.

We found no overt anatomical differences between Z-VAD-fmk and control embryos (compare Figures 9A,A′,A″ with B,B′,B″), except perhaps a negligible decrease in size under treatment conditions. Similarly, on first inspection, JNK inhibition alone vs. SP600125 + Z-VAD-fmk inhibitor appeared to generate similar phenotypes (compare Figures 9C,C′,C″ with D,D′,D″). However, both the nerve cord and notochord appeared more regular and extended, and cCasp-3 expression was reduced in the double-treated embryos, both quantitatively (fewer patches) and qualitatively (lower intensity) (see insets u–w and x–z), suggesting that Z-VAD-fmk might provide some mild rescue of the Caspase-dependent apoptosis induced by SP600125. In addition, although both conditions resulted in rounded cells accumulating within the gut space, as well as lateral bulges of rounded actin-rich cells between the somites and endoderm (insets v and y), many fewer of these were pycnotic, with the characteristic central actin aggregates, particularly within the anterior gut when both inhibitors were used (compare closed and open magenta and blue arrowheads in w and z, respectively). Indeed, these cells appear to have rounded up and exited their tissues, but not to have died. Taken together, the mild rescue seen in these experiments suggest that the *orca* phenotype is probably the result of both the effect of JNK inhibition on polarity/cell movements and on the normal programmed cell death required to remodel amphioxus tissues during early development.

### MAPK/ERK Signalling Is Ectopically Activated

**FIGURE 10:**
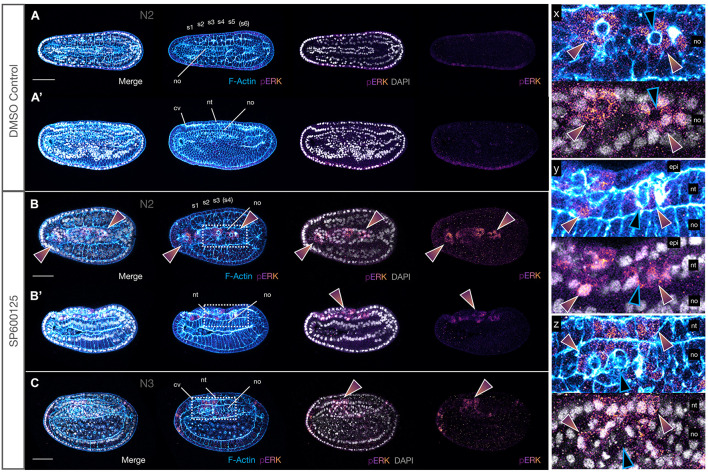
JNK inhibition leads to upregulation of pERK in neighbours of extruding cells. **(A,A′)** N2 stage DMSO control embryo with five formed somite pairs. The notochord cells are arranged in two parallel rows, except anteriorly under the cerebral vesicle, and are multiple cell layers thick. There is little to no pERK staining. **(B,B′)** SP600125-treated embryos allowed to progress to the same stage have broader monolayered notochords. They accumulate cytoplasmic and nuclear pERK in notochord (rainbow arrowheads in **B,B′** and inset y) and overlying neural and epidermal cells (rainbow arrowheads in **B** and inset x). Two different embryos are shown. **(C)** This pattern is seen in treated embryos allowed to progress to the equivalent of the N3 stage, in which notochord cells appear to attempt to stack dorsoventrally in multiple layers (inset z). In all cases, pERK accumulation occurs in cells neighbouring rounding cells apparently in the process of extruding (note apical rounding and F-Actin accumulation in membranes, blue outlined black arrowheads in insets x, y, and z). Phalloidin staining of F-Actin is in blue and phosphorylated ERK (pERK) immunoreactivity is in a purple/orange gradient, while DAPI stained nuclei are in grey. Embryos are orientated with anterior to the left either dorsal (**A,B** and inset x) or lateral views (**A′,B′,C** and insets y and z) are shown. Representative embryos are shown. cv, cerebral vesicle; epi, epidermis; nt, neural tube; no, notochord; pharynx, ph; pp, preoral pit; s1, s2, etc., somite pairs. Scale bars = 50 μM.

Having ascertained that JNK inhibition leads to an apparent delamination and death of misplaced cells in several tissues, resulting in CE defects, we wanted to determine the mechanism by which this might occur. Several reports suggest that JNK interacts with several pathways including MAPK and TGFβ/BMP to coordinate cellular movements, cell division and polarity (Gros et al., 2010). Specifically, regulation of MAPK–ERK activity governs cellular behaviour and extrusion, followed in many cases by apoptosis, in vertebrate and invertebrate models (Moreno et al., 2019; Aikin et al., 2020; Tada, 2021). SP600125 is a highly selective inhibitor of c-Jun phosphorylation but has no observable effect on ERK or p38 phosphorylation (Bennett et al., 2001). Thus, any changes in ERK phosphorylation act as a readout of MAPK signalling, and we therefore reasoned that this pathway might be responsible for some of the altered cellular behaviour and patterning that we observed. We therefore assessed levels of pERK in SP600125-treated N2 stage embryos, prior to the onset of the most dramatic defects caused by JNK inhibition, using a pERK antibody that detects active ERK signalling. Our results indicate that under control conditions, there was very little localised expression of activated MAPK (Figures 10A,A′). However, in embryos treated with the JNK inhibitor, we found strong ectopic expression in epidermal cells, and in a few cases, in neural cells (Figure 10B′ and inset y). More dramatically, we saw an unexpected upregulation of pERK in many medial notochord cells, but also in anterior notochord cells (Figure 10B and inset x, white outlined rainbow arrowheads), specifically in cells neighbouring the rounding cells, whose membranes are F-Actin rich, and which appear to be targeted for extrusion (Figure 10B and inset x, blue outlined black arrowheads). This response was also apparent at later stages (N3) when most cell rearrangement occurs within the notochord (note multi-layered aspect) and apoptotic cells have accumulated within the gut (Figure 10C and inset z). Taken together, these data suggest that there is a correlation between loss of JNK-mediated polarity, cellular extrusion and pERK activation, and that this response is non-cell autonomous in the chordamesoderm/notochord.

## Discussion

Although pharmacological inhibition of JNK activity using SP600125 is commonplace in vertebrate cell lines or epithelia *in vitro* (Yu et al., 2019; Brunt et al., 2021), studies on whole embryos are comparatively rare. This is surprising given its versatility as a tool, particularly in aquatic organisms with external development, where it allows studies of morphogenetic processes of complex tissues within specific temporal windows, without the need for designing labour and time-intensive tools. This is true even in models with long-established genetic manipulation methodologies such as zebrafish (e.g., He et al., 2016; Li et al., 2019), or even ascidians (e.g., Krasovec et al., 2019), especially where late functions need to be assessed. In amphioxus, treatment with SP600125 results in a complex phenotype likely encompassing the full range of its functions, suggesting it is a good chordate model for understanding the evolution of JNK function at the invertebrate to vertebrate transition.

### Evolution of the Chordate Functional JNK Repertoire

The two whole genome duplications that occurred in the vertebrate ancestor (Dehal and Boore, 2005) resulted in the presence of multiple paralogues in vertebrate relative to invertebrate chordate genomes, barring any lineage-specific duplication or gene loss events. The evolution of JNKs represents a near textbook-case scenario, with single *jnk* genes in amphioxus and ascidians (similarly to other invertebrates, e.g., *basket* in *Drosophila*; Satou et al., 2003; Bertrand et al., 2009), and three orthologues in mammals (Zeke et al., 2016). In parallel, the mitogen activated signalling cascade upstream of JNK is considerably more simplified in invertebrate chordates relative to vertebrates (Satou et al., 2003; Bertrand et al., 2009). In vertebrates, such duplication events have conferred some genetic redundancy, protecting critical cellular functions. For instance, single *JNK* mutant mice survive, but double mutants for *JNK1* and *JNK2* are embryonic lethal, with region-specific dysregulation of apoptosis in the early brain (Kuan et al., 1999). Apparently, some constraint exists on the number of JNK copies tolerated in vertebrate genomes. Bony fish, for instance, might be expected to harbour many more duplicates (at least 8, assuming no gene losses) due to an independent genome duplication in the teleost ancestor (Glasauer and Neuhauss, 2014). Surprisingly, however, a single *JNK2* and *JNK3*, and only two *JNK1* paralogues named *JNK1a* and *JNK1b* have been identified in zebrafish (Santos-Ledo et al., 2020). Similarly, *Xenbase* reports only two *MAPK8/JNK1*, one *MAPK9/JNK2*, and two *MAPK10/JNK3* genes in *Xenopus laevis* (Karimi et al., 2018). This is still fewer than expected but can be explained by the species’ allotetraploid origins, likely reflecting asymmetric evolution of the subgenomes, with one chromosome set more prone to gene loss (Session et al., 2016). To compensate, alternative splicing of *jnk*s appears to be rampant in vertebrates, increasingly dramatically the functional repertoire of JNK proteins. For example, in zebrafish, four isoforms have been identified for each of the JNK1a and JNK1b paralogues, with the *jnk1a Ex7 Lg* transcript required to generate correct numbers of cardiac progenitors within the ventricle of the developing heart (Santos-Ledo et al., 2020). In humans, all three JNK genes encode multiple alternative splice isoforms: four each for JNK1 and JNK2, and eight for JNK3, of which only three have been characterised (Zeke et al., 2016). The isoforms include alternative exons in the C-terminal lobe of the kinase domain (α- and β-isoforms arising from use of a mutually exclusive exon pair, normally the sixth, but nomenclature is inconsistent), and in both the N- and C-terminal flexible extensions of the proteins. Functionally, it is predicted that different JNK isoforms will exhibit different kinetics and enzyme activities as well as substrate affinities (Zeke et al., 2016). Intriguingly, Ait-Hamlat et al. (2020) have recently predicted the existence of a new JNK1 isoform lacking exons 6, 7, and 8, the critical region for kinase structural stability and MKP7 phosphatase binding, which they named JNK1δ. Nevertheless, the isoform has stable secondary structure in solution, is expressed in human, mouse and fugu, and produces four peptides (Ait-Hamlat et al., 2020). Mechanistically, splicing of JNKs is regulated by Nova through differential availability of Nova-binding sites in the paralogues (Letunic et al., 2002; Zeke et al., 2016), and Nova also regulates JNK splicing in amphioxus in a tissue-specific fashion (Irimia et al., 2011). These studies highlight the increased functional complexity possible through both duplication and alternative splicing, and help explain why it is difficult to dissect the myriad roles of JNK signalling even in amphioxus, which has a single gene. It will be informative to further study differences between amphioxus and vertebrates in function of JNK paralogues vs. isoforms during development through more targeted approaches.

### JNK Plays a Conserved Role in Gastrulation and Convergence Extension Movements

This study adds to several lines of evidence pointing to a conserved role for JNK signalling in regulating tissue movements, both during gastrulation and in processes intimately linked to axis elongation. For instance, our gastrulation arrest phenotype, with little to no change in cell type specification, closely parallels that seen in sea urchins treated with SP600125, which inhibited invagination, but not endoderm differentiation (Long et al., 2015). Although our understanding of early patterning processes in amphioxus has advanced dramatically in the last few years (Li et al., 2017; Aldea et al., 2019; Kozmikova and Kozmik, 2020; and many others), still little is known about the invagination process at the cellular level. While we focus here on the role of JNK in later processes, it is intriguing that we found no evidence of inductive events between endoderm and ectoderm, since disruption of archenteron formation and lack of normal apposition of endoderm and ectoderm resulted in no changes in gene expression; notochord, mesoderm and neural tissues all appear properly specified in early treatments, at least until late gastrula stages.

Intimately linked to axis elongation in chordates is the process of notochord morphogenesis, which like invagination requires precise spatio-temporal control of cellular behaviours. In vertebrates such as zebrafish and *Xenopus*, correct CE movements of dorsal mesoderm at the midline require JNK signalling downstream of Wnt/PCP pathways (Yamanaka et al., 2002; Kim and Han, 2005; Williams and Solnica-Krezel, 2020), although there is considerable variation in how mediolateral intercalation behaviour is regulated across species (Keller and Sutherland, 2020). Nevertheless, our results suggest conservation of the core polarity mechanism in chordates, since inhibition of JNK activity causes dramatic defects in medial chordamesoderm and notochord cells, in spite of apparent correct specification of midline structures (e.g., *Brachyury2* and *chordin*), and a strong axial elongation phenotype. Intriguingly, this also translates to a truncation of the anterior notochord, correlating with a reduction in *six3/6* expression in a notochordal domain rostral to the hypothalamo-prethalamic primordium of the cerebral vesicle (“HyPTh”; Albuixech-Crespo et al., 2017). Despite several differences with vertebrate anatomy and genoarchitecture, this *six3/6*^+^ region may represent the amphioxus homologue of the prechordal plate (Albuixech-Crespo et al., 2017). In vertebrates, notochord formation from axial mesoderm requires the complex orchestration among active migration of prechordal cells (Bosze et al., 2020), non-canonical Wnt11-mediated cellular movements of paraxial mesoderm (Heisenberg et al., 2000), and CE of deep neural cells (Keller and Sutherland, 2020). Treatment with SP600125 also inhibits the mediolateral cell intercalation of the neurectoderm required for axial elongation in the annelid *Platynereis dumerilii* (Steinmetz et al., 2007). Thus, inhibition of JNK throughout amphioxus tissues likely perturbs cellular polarity and coordination of behaviours across germ layers, culminating in the truncated axis phenotype we see here, highlighting an ancient global function for this pathway in regulating CE movements.

### JNK Is Required for Formation of Oral Structures in Amphioxus

Our results show that inhibiting JNK signalling has a profound and specific effect on the formation of anterior and oral structures in amphioxus, beyond those attributed to anterior notochord extension defects. For instance, we show that even mild treatments with SP600125 result in loss of the ciliated preoral pit, derivative of the initial kidney (or Hatschek’s left diverticulum) and by extension cells of the posterior first left somite (Holland, 2018). This may be due to a loss of migratory activity of precursors detaching from the somite, and/or to degradation of the somite itself. We also fail to see any of the oral perforations in pharyngeal endoderm that presage club shaped gland, gill slit or mouth formation. In fact, cells appear to continue to round up and delaminate from anterior ventral endoderm until very late developmental stages (e.g., Supplementary Figure 4). Although there is continued debate surrounding the homology of chordate mouths (reviewed in Holland, 2018), the conservation of a role for JNK in perforation at the junction of epithelial membranes is consistent with our observations. In *Xenopus*, JNK signalling is crucial for epidermal integrity and E-Cadherin localisation at the adherens junction, with a failure of buccopharyngeal opening and subsequent embryonic mouth formation in embryos treated with SP600125 or injected with morpholinos against *JNK1* (Houssin et al., 2017). However, this phenotype was not caused by either the apoptotic or cell cycle regulatory functions of JNK signalling (Houssin et al., 2017). Because our treatments were continuous rather than targeted to the specific window of pharyngeal differentiation, we cannot exclude the possibility that other factors are at play. Indeed, even embryos treated with only 2.5 μM SP600125, with or without Caspase inhibition, continue to show large numbers of rounded cells apparently delaminating within the pharyngeal region. This is also consistent with a more general function in adhesion or anterior endoderm specification, similarly to the role played by Wnt/JNK signalling in determining foregut identity and cellular morphology in zebrafish (Zhang et al., 2016) and endodermal tissue integrity during gut elongation in *Xenopus* (Dush and Nascone-Yoder, 2013). The apparent loss of the anterior ventral endoderm expression domain of *wnt11* might be consistent with a loss of specific structures in this region. Future work should be aimed at specifically addressing the link between JNK signalling and morphogenesis of oral and “head” structures, including the expression of late endoderm markers, in cephalochordates.

### Wnt Genes May Be Transcriptional Targets of JNK Signalling in Amphioxus

In several cellular contexts, the non-canonical Wnt/PCP pathways are mediated by JNK *via* specific Wnt ligands, independently of nuclear β-catenin-dependent canonical Wnt signalling. As previously discussed, Wnt/JNK signalling through Wnt5 or Wnt11 paralogues plays an important role during avian, zebrafish and *Xenopus* gastrulation (Heisenberg et al., 2000; Hardy et al., 2008; Seo et al., 2010; Keller and Sutherland, 2020). Wnt5a is also responsible for correct orientation of mesenchymal cell movements and division during proximo-distal limb bud elongation in chick and mouse through JNK signalling (Gros et al., 2010). However, Wnt genes may also themselves be targets of JNK activity. For instance, depletion of JNK activators MKK4b and MKK7 results in CE defects during gastrulation and abnormal somitogenesis in zebrafish, with *wnt11* a direct downstream target of Wnt/JNK signalling (Seo et al., 2010).

In amphioxus, the functional data permitting the identification of non-canonical Wnt ligands are still lacking, and comparative studies of the Wnt family expression atlas across chordates suggest that considerable function shuffling of Wnt ligands has occurred in these lineages (Somorjai et al., 2018). In particular, the expression of *wnt2* in the amphioxus cerebral vesicle/nerve cord is conserved with vertebrates but is a cephalochordate novelty in the notochord (Somorjai et al., 2018). Nevertheless, our data suggest that *wnt2* and *wnt11* may be (direct or indirect) targets of a non-canonical Wnt/JNK signalling pathway in amphioxus within specific spatio-temporal contexts. For instance, *wnt11* appears to be ectopically activated in the posterior ectoderm after SP600125 treatment, which is consistent with the zebrafish data (Seo et al., 2010). However, it is also downregulated in the paraxial mesoderm and in the chordoneural hinge, and possibly in the ventral endoderm. This could be explained if Wnt11 is both upstream of JNK signalling and its target, with SP600125 resulting in threshold-dependent autoregulation in a negative feedback loop. This is not unheard of, as we have shown similar contradictory downregulation of *sp5* in the amphioxus tailbud in response to strong and prolonged treatment with a GSK3-β inhibitor (which activates Wnt/β-catenin signalling), when otherwise it behaves as a positive target (Dailey et al., 2017). *Wnt2*, in contrast, appears to be downregulated persistently and exclusively in the notochord, with no effect of the inhibitor on expression in the nerve cord, suggesting *wnt2* may be a direct target of JNK signalling in chordamesoderm. However, we cannot currently exclude a second possibility, which is that the loss of *wnt2* and *wnt11* expression reflects an indirect effect of JNK on notochord or endoderm differentiation, respectively. In vertebrates, Wnt2, Wnt5a/5b, and Wnt11 induce differentiation of human cardiomyocytes from lateral plate mesoderm *via* an atypical Wnt/Ca^2+^ pathway (Mazzotta et al., 2016). There is also increasing evidence for direct crosstalk between JNK and the canonical Wnt signalling pathways at the transcriptional level (Bikkavilli and Malbon, 2009), complicating interpretation. Because of its late onset of expression, *Wnt2*^–/+^ or *Wnt2*^–/–^ transgenics could conceivably be generated in amphioxus to help distinguish between these possibilities.

### Changes in Caspase-3-Mediated Apoptosis and Proliferation Within Tissues Cannot Explain SP600125-Induced Cellular Loss

It is now well established (if not well understood) that JNK plays important pro-proliferative and pro-apoptotic roles during development and tissue replacement, depending on the cellular context (reviewed in Ricci and Srivastava, 2018; Pinal et al., 2019; Guerin et al., 2021). JNK activity has also been proposed to act as a pivot point, balancing cell death and proliferation to maintain body proportions during whole body regeneration and starvation-induced homeostatic degrowth in flatworms (Almuedo-Castillo et al., 2014). Our *orca* phenotype could therefore be interpreted as a dysregulation of this equilibrium, resulting from either constitutive or local increases in apoptosis or, alternatively, decreases in proliferation.

In amphioxus, TUNEL staining – an assay used to detect apoptotic DNA cleavage as well as other types of DNA damage – previously suggested that programmed cell death is regulated during embryogenesis, with apoptosis likely required for anterior endoderm remodelling starting during neurulation stages (Bayascas et al., 2002). At larval stages, dying cells are scattered throughout the tissues, with concentration of signal in the pharyngeal and tail regions (Bayascas et al., 2002), and a burst is again seen at the onset of metamorphosis around the forming mouth, gills and anus, in the club shaped gland and preoral pit, and in tail epidermis (Holland et al., 2009). Tail regression, on the other hand, appears to primarily employ remodelling (Koop et al., 2011). In contrast, in the solitary ascidian *Ciona*, programmed cell death is necessary for regression of the tadpole tail and settlement (Krasovec et al., 2019). Here, signalling by both ERK and JNK MAP kinases is required (Chambon et al., 2007), and tail regression fails when either the pathway is inhibited, or Caspase activity is blocked (Chambon et al., 2002; Krasovec et al., 2019).

Amphioxus is known to have a single Caspase-3/7 orthologue of mammalian effector caspases with similar substrate preference to vertebrate Caspase-7, and which induces apoptosis. Its transcript expression is rather non-specific within mesendoderm and uncorrelated with TUNEL; the authors conclude that the role of Caspase-3/7 is to confer the ability for cells to die rather than instructing apoptosis (Bayascas et al., 2002). Similarly, in control embryos we see little convincing immunoreactivity with cCasp-3 antibody, which marks cells that are committed to apoptosis. In contrast, SP600125 induces considerable nuclear fragmentation coincident with expression of cCasp-3. However, cCasp3^+^ cells are rarely found within the embryonic tissues, being generally limited to cells that have already clearly delaminated into luminal or coelomic spaces (e.g., within the archenteron or between the epidermis and neural tube), and only within specific early developmental windows corresponding to neurula stages. Therefore, we do not see a pattern of Caspase-3 activity corresponding to previously reported TUNEL staining. This suggests that other cell death pathways (possibly including necroptotic; Tait and Green, 2008) may be involved during normal development. Consistent with this, single treatments with the pan-Caspase inhibitor Z-VAD-fmk, which was demonstrated to inhibit Caspase-3/7 from cleaving its substrates *in vitro* (Bayascas et al., 2002), had little effect on morphogenesis. However, it did appear to rescue to some degree the Caspase-3-mediated apoptosis of cells that had already delaminated into luminal spaces. Although we have no lineage data to support this (indeed, we never saw gene marker expression within delaminated cells, suggesting it was lost once cells became mis-specified, or that they had already progressed within the apoptotic pathway), our observations suggest that some of these cells derive from the anterior or pharyngeal endoderm, which might be primed to undergo programmed cell death. Unfortunately, it was beyond the scope of this work to perform TUNEL staining in our SP600125 or SP600125 + Z-VAD-fmk rescue experiments, but we predict that the pattern of cell death would differ relative to controls specifically within anterior ventral endoderm. Thus, JNK inhibition may not directly activate Caspase-3-mediated apoptosis within amphioxus tissues proper, which is instead induced secondarily within “shed” cells. An alternative, which we do not favour, is that cCasp-3 immunoreactivity is simply not sensitive enough in early stages of delamination to detect in our experiments.

Regardless of how cells are lost from tissues, changes in the levels or patterns of proliferation relative to baseline might in part account for the phenotypes we observe. Under control conditions, we saw broad but variable patterns of cell division, with relatively large numbers of PH3^+^ cells in the endoderm throughout development. Few cells were labelled in the nerve cord, somites, or notochord in any one individual, with more consistent proliferation in the posterior notochord and nerve cord within the tailbud. Our results are broadly congruent with BrDU pulse-chase experiments (Holland and Holland, 2006), which label dividing cells and their progeny, demonstrating that much of the raw material of the notochord is generated by proliferation in the gastrula and early neurula, with proliferation gradually declining at later stages except in the anterior and posterior tips. Although it was too challenging to accurately quantify proliferation in treated embryos here, these patterns appear to be maintained after exposure to SP600125, and therefore are unlikely to account for shortening of the primary axis. However, we cannot discount the possibility that JNK inhibition might in fact delay or arrest passage through the cell cycle, and thus contribute to defects in elongation and patterning. For instance, treatment with SP600125 prolongs mitotic progression through S and G2 phases of the cell cycle in synchronised mouse cells; such growth-inhibited cells eventually die *via* apoptosis (Du et al., 2004). SP600125 also enhances Caspase-3 dependent apoptosis in tumour cells (Kuntzen et al., 2005) and prevents transition from the G2 to M phase (Kuntzen et al., 2005; Kim et al., 2010). However, this arrest may occur *independently* of its ability to inhibit JNK, since specific knockdown of *JNK1/2* by siRNA has no effect on PH3 protein levels in cells (Kim et al., 2010). These studies might also help to explain some apparently contradictory results in flatworms, which show both that SP600125 causes G2/M arrest (Tasaki et al., 2011) and faster entry of neoblasts into mitosis after *JNK(RNAi)* (Almuedo-Castillo et al., 2014). Our static snapshots using PH3 immunostaining, which only labels cells in G2/Mitosis, clearly do not capture the complexity of cell cycle dynamics. Nevertheless, even if there is some non-specific effect of SP600125 on proliferation kinetics in our experiments, or a general delay in cell division, we consider it unlikely that a decrease in proliferation alone can account for our results.

In light of these observations, we currently favour the hypothesis that inhibiting JNK signalling tips the balance in favour of cellular loss, resulting in shortened and mis-proportioned embryos, with cell division unable to compensate; apoptosis is effectively a secondary effect of a loss of cellular polarity and tissue integrity. However, careful analysis of cell cycle dynamics and cellular behaviours of labelled cells *in vivo*, as well as more detailed studies on JNK-mediated cell death in specific tissues using molecular markers, will be required to test and refine this model to dissect the context-dependency of apoptosis and proliferation during amphioxus morphogenesis.

### Phosphorylated ERK Activation May Be a Mechanism for Cellular Extrusion in Notochord and Neural Cells

Our observation that JNK inhibition appears to cause cytoskeletal changes, rounding, and delamination of cells into extracellular spaces, combined with CE-type defects, might suggest that active cellular extrusion of mis-specified cells is occurring, followed by (or concomitant with) their Caspase-3-mediated apoptosis. Both live and apoptotic cellular extrusion are important regulators of growth and cell density and are critical for maintaining tissue homeostasis (Ohsawa et al., 2018). The orientation of cellular extrusion – either basally or apically depending on the model – can lead to either hypertrophic overgrowth (cancer) or apoptosis. Importantly, epithelial extrusion is a highly dynamic process *in vivo*, requiring coordination of the actin cytoskeleton and cell junction dis/assembly in cells fated to die as well as their neighbours to maintain tissue integrity (reviewed in Gagliardi and Primo, 2019). Intriguingly, mechanical stress due to crowding alone within a proliferating tissue is also sufficient to cause Caspase induction and basal delamination in the fly notum and may depend on the direction of cell division (Levayer et al., 2016; reviewed in Tada, 2021). Thus, various types of signal can lead to cell extrusion when cells are stressed or dying.

The role of JNK in these processes is complex, and results are often contradictory depending on the cellular context. Typically, inappropriate JNK induction in “loser cells” is associated with their cellular elimination and apoptosis in a cell competition scenario, but can also result in hyperproliferation, for instance during regeneration (Pinal et al., 2019). It has also been suggested that cell cycle progression in G2/M is a checkpoint for cellular extrusion, such that “unfit” cells die if they are in G2 during cell competition (Tada, 2021). In the context of our work in amphioxus, SP600125 treatment might lead to extrusion of cells in several ways, including changes in apicobasal polarity or orientation of cell division, cell crowding, and competition. These might be particularly prevalent in tissues undergoing active cellular rearrangement and extension, such as the notochord at mid-neurula stages, or that proliferate and remodel extensively, such as the anterior ventral endoderm.

One of the key findings of this study was that after JNK inhibition, ERK signalling is activated in the neighbours of cells undergoing extrusion and dying, as evidenced by instances of nuclear fragmentation and Caspase-3 cleavage. This phenomenon was most apparent in the monolayer of the early notochord, but pERK^+^ cells were also sometimes seen in the nerve cord. Our data are both consistent with and appear to contradict results in other systems. For instance, in zebrafish, correct regulation of ERK activity is important for axis elongation and notochord differentiation from chordamesoderm, but not for its early patterning or specification (Hawkins et al., 2008), similarly to amphioxus and contrary to its early role in notochord determination in *Ciona* (Minokawa et al., 2001; Sakabe et al., 2006). U0126 treatment (which blocks ERK1/2 phosphorylation and thus ERK activation), however, results in an increase in TUNEL^+^ apoptotic cells in 24hpf zebrafish, but not in the notochord (Hawkins et al., 2008), which is inconsistent with our own results. In a similar vein, during *Ciona* tail regression, ERK activity induces apoptosis in the notochord and epidermis (Chambon et al., 2002, 2007). However, SP600125 or U0126 treatment both reduce the number of TUNEL^+^ cells in the tail. Chambon et al. (2007) propose a model whereby normal JNK activation in the CNS induces apoptosis through activation of ERK in adjacent tissues, including the notochord, *via* modification of the ECM. The endoderm and CNS themselves are non-receptive and escape the death signals (Chambon et al., 2002, 2007). We would argue that some of these apparent discrepancies across studies may be due to regulative vs. determined developmental mode, the level of cellular resolution, variable tissue-specific and temporal responses, or differences between ERK and JNK targets. It is of further note that in the *Drosophila* epidermis, mis-specified cells exhibit aberrant EGFR signalling with ERK activation, leading to apoptosis, whereas endogenous ERK activation apparently promotes cell survival (Crossman et al., 2018), and that cumulative ERK dosage impacts developmental income (Johnson and Toettcher, 2019). Thus, ERK response varies both across physiological conditions as well as under different aberrant ones.

Several lines of evidence suggest that regulation of ERK might also link cellular extrusion, cell competition, and cellular survival outcomes with mechanical forces within tissues (Moreno et al., 2019; Valon et al., 2021). Compaction and/or a reduction in tension results in down-regulation of EGFR/ERK signalling in looser cells, leading to their elimination *via* apoptosis; conversely local tissue stretching was correlated with activation of ERK (Moreno et al., 2019). Interestingly, compression in the *Xenopus* embryonic epithelium by high gravity causes the cells to activate FGFR/ERK signalling by sensing tensile forces at the adherens and tight junctions (Kinoshita et al., 2020). While we did not directly manipulate forces, it seems likely that SP600125-treated embryos are subjected to considerable compressive forces and tension due to incompatible changes in growth and cellular behaviours across different tissues during neurulation and gastrulation. These conflicts might be expected to be strongest in the notochord as it fails to elongate properly and cells compete for space, and in the anterior somites as they continue to be compressed by cellular accumulation. Punctate β-catenin accumulation at the membranes around extruding (normally pERK low) cells might provide some limited evidence to support an effect on the adherens junctions in the notochord. The fact we saw no convincing pERK modulation in anterior endoderm cells, which appear to also round up and delaminate, can be explained if the effect of JNK inhibition here were due to changes in the plane of division symmetry during proliferation or the cell cycle, rather than from mechanical stresses associated with the highly regulated requirements for CE seen in notochord. Although few specific antibodies are available in cephalochordates, it would be interesting to further examine the effects of Wnt/JNK and other pathways known to affect cellular movements or polarity (e.g., ROCK) on both ERK activity and distribution of apicobasal membrane proteins.

### Perspectives

This research presents the first data supporting a putative role for Wnt/JNK signalling in cell behaviour and cell fate decisions during cephalochordate development. Specifically, treatment with the SP600125 inhibitor causes axis shortening consistent with CE defects, accompanied by dramatic changes in the notochord both at the cellular and the molecular levels. Anterior endoderm patterning and somite derivatives are also affected by inhibition of JNK, hinting at some conserved mechanisms with vertebrates, in addition to more ancient roles in regulating the balance between apoptosis and cell survival. Combining the benefits of invertebrate (e.g., a single orthologue) and vertebrate models (chordate body plan and morphogenesis), amphioxus is therefore a good system for studying the myriad and complex roles of JNK signalling in cell biology and morphogenesis.

Our work has several limitations, including the reliance on fixed material and therefore static snapshots of highly dynamic processes *in vivo*. It remains difficult to effectively immobilise live amphioxus embryos, which at all stages are extremely fragile. The ciliary movement embryos use to glide through the water column, even prior to active muscle contraction-dependent swimming in larval stages, has proven difficult to pharmacologically inhibit without causing other defects. Even trying to hold embryos in place in agarose, without applying undue pressure using coverslips – which can itself modulate normal cellular behaviours and even gene expression – is short lived as the embryos ultimately rotate in place. In addition, although the reliance on pharmacological treatment presents notable advantages, it does prevent the dissection of the molecular mechanisms underlying the context-dependent functions of JNK achievable with more targeted genetic approaches. However, the generation of transgenic or mutant lines (e.g., Li et al., 2017), for example to allow temporal and spatial analysis of cell movements at high resolution, is still limited to a handful of laboratories with access to tropical species with short life cycles, and the resources to maintain breeding animal colonies long term. These considerations apply to many non-traditional, particularly marine model systems. Nevertheless, advances such as hydrogel encapsulation (Burnett et al., 2018) or acoustic trapping combined with live imaging (Yang et al., 2019) have shown proof of principle that embryos can be maintained alive and develop normally to allow studies of cellular movements. Combined with better fluorescent dyes, for instance to label cell membranes, and fast 3D imaging, as well as further developments in cell tracking, imaging dynamic cells movements in amphioxus should soon prove more tractable, permitting more detailed analyses of the evolution of morphogenetic and cellular processes at the origin of chordates.

## Data Availability Statement

The original contributions presented in the study are included in the article/Supplementary Material; further inquiries can be directed to the corresponding author.

## Author Contributions

IMLS conceived the study, performed the experiments, analysed the data, provided resources, and wrote the manuscript. MTE modelled SP600125 and JNK protein binding and contributed to Figure 1. HE and JG-F provided resources. All authors read and approved the final manuscript.

## Conflict of Interest

The authors declare that the research was conducted in the absence of any commercial or financial relationships that could be construed as a potential conflict of interest.

## Publisher’s Note

All claims expressed in this article are solely those of the authors and do not necessarily represent those of their affiliated organizations, or those of the publisher, the editors and the reviewers. Any product that may be evaluated in this article, or claim that may be made by its manufacturer, is not guaranteed or endorsed by the publisher.

## Footnotes

1
https://blast.ncbi.nlm.nih.gov/

2
https://metazoa.ensembl.org/

3
https://www.ebi.ac.uk/Tools/msa/clustalo/

## Supplementary Material

Supplementary Figure 1 Alignment of three JNK sequences from human (MAPK8, 9, and 10) and single orthologues from amphioxus species *Branchiostoma floridae*, *Branchiostoma lanceolatum*, and *Branchiostoma belcheri*. Transcriptomic data were used to manually correct errors in *B. lanceolatum* gene model BL14252 from Ensembl to generate a consensus sequence. The alignment was generated in Seaview using Muscle.

Supplementary Figure 2 Defects in invagination and developmental arrest during gastrulation after SP600125 treatment (5 μM) at blastula stage. Exogastrulae arrested during invagination by JNK inhibition are boxed in yellow. Column 1 embryos were stopped at the gastrula stage (control to left). Lateral (top) and blastoporal (bottom) views are shown for each marker gene. Columns 2 and 3 embryos were stopped at neurula stages (control to right). Lateral (top) and dorsal (bottom) views are shown for each marker gene. In some cases blastoporal views are shown (*dll*, *nodal*, and *neurogenin*). Wild type gastrula stages are shown on the left for comparison to show how gene expression resembles earlier stages of development. For *dkk1/2/4*, treated embryos allowed to further develop are boxed in cyan, showing no change in gene expression relative to those stopped earlier. Scale bars = 50 μM.

Supplementary Figure 3 Additional markers used to characterise the *orca* phenotype show inconclusive results or no change in expression relative to controls. Embryos were treated with SP600125 as per Figure 2. Lateral views show anterior to the left and dorsal up. Dorsal views show anterior to the left. Dorsal views for *otx* are omitted as they were uninformative (the underlying endodermal expression was too strong, masking the neural domains). The black outlined arrowhead highlights loss of separation between neural plate and endoderm expression domains of *otx* in treated embryos. The white asterisk in *wnt4* shows non-specific staining caused by particulate material stuck to the embryo surface. Generally, embryos were stained longer than required to ensure all real domains became revealed, resulting in some general background (e.g., bluish/purplish tinge, especially in mesendoderm, in both treated and controls). Scale bars = 50 μM.

Supplementary Figure 4 Limited apoptosis in late embryos after weak JNK inhibition. **(A,A′,A″)** T0 stage DMSO control embryo (stitching of two separate confocal images). Perforation of the pharynx is imminent (yellow asterisks) but little cleaved Caspase-3 is evident. **(B,B′,B″)** Stage matched 2.5 μM SP600125 treated embryos have a disorganised but differentiated central notochord and lack anterior structures, including the pharynx (yellow asterisks), which consists of many rounding cells ventrally (blue arrowheads). The gut contains cleaved Caspase-3-positive pycnotic cellular material (**B″**, insets x, x′ and y, y′), and occasional notochord cells are apoptotic and in the process of extrusion (white arrowheads in insets x, x′ and y, y′). Overall, however, there is little evidence of Caspase-3-dependent apoptosis at these late stages. Phalloidin staining of F-Actin is in blue, cleaved Caspase-3 (cCasp-3) immunoreactivity is in magenta, and DAPI stained nuclei are in grey. nt, neural tube; cv, cerebral vesicle; no, notochord; ph, pharynx. Embryos are orientated with anterior to the left and dorsal up. Scale bars = 50 μM.

Supplementary File 1 Sequences of new probes used in this study.
