# Supplementary material for: JNK Mediates Differentiation, Cell Polarity and Apoptosis During Amphioxus Development by Regulating Actin Cytoskeleton Dynamics and ERK Signalling
Source: Front Cell Dev Biol. 2021 Oct 29;9:749806. doi: 10.3389/fcell.2021.749806 (PMC8586503; doi:10.3389/fcell.2021.749806)

Supplementary Figure 1. Alignment of three JNK sequences from human (MAPK8, 9, 10) and single orthologues from amphioxus species *Branchiostoma floridae*, *Branchiostoma lanceolatum* and *Branchiostoma belcheri*. Transcriptomic data were used to manually correct errors in *B. lanceolatum* gene model BL14252 from Ensembl to generate a consensus sequence. The alignment was generated in Seaview using Muscle.

```
                     1
Hsa_MAPK8            ---------- ---------- ---------- ---------- MSRSKRDNNF YSVEIGDSTF TVLKRYQNLK PIGSGAQGIV CAAYDAILER NVAIKKLSRP
Hsa_MAPK9            ---------- ---------- ---------- ---------- MSDSKCDSQF YSVQVADSTF TVLKRYQQLK PIGSGAQGIV CAAFDTVLGI NVAVKKLSRP
Hsa_MAPK10           --MSLHFLYY CSEPTLDVKI AFCQGFDKQV DVSYIAKHYN MSKSKVDNQF YSVEVGDSTF TVLKRYQNLK PIGSGAQGIV CAAYDAVLDR NVAIKKLSRP
Bflo_XP_035669587    ---------- ---------- ---------- ---------- ---------- ---------- ---------- ---------- ---------- ----------
Bflo_XP_035669588    ---------- ---------- ---------- ---------- ---------- ---------- ---------- ---------- ---------- ----------
Bflo_GETA01026605    MHRSPSWLLK SFEVELDLRL AFSQAFCDAV DLTYLAQHYR MSKLAANSQF YTVEVGDSTF TVLKRYQNLK PIGSGAQGIV CAAYDTLTGQ NVAIKKLSRP
Blan_BLAG15101005    ---------- ---------- ---------- ---------- MSKLAANSQF YTVEVGDSTF TVLKRYQNLK PIGSGAQGIV CAAYDTLTGQ NVAIKKLSRP
Blan_BL14252         MHRSPSWLCK SSEVEFDLRL AYCQAFCDAV DPTHLAQHFR MSKLAANSQF YTVEVGDSTF TVLKRYQNLK PIGSGAQGIV CAAYDTLTGQ NVAIKKLSRP
Blan_JT868948        ---------- ---------- ---------- ---------- ---------- ---------- ---------- ---------- ---------- ----------
Blan_consensus       MHRSPSWLCK SSEVEFDLRL AYCQAFCDAV DPTHLAQHFR MSKLAANSQF YTVEVGDSTF TVLKRYQNLK PIGSGAQGIV CAAYDTLTGQ NVAIKKLSRP
Bbel_XP_019639469    MHRSPTWFYK SFEVEFDLRL AFSQAFCDAI DLTHLAQHFR MSKLAANSQF YTVEVGDSTF TVLKRYQNLK PIGSGAQGIV CAAYDTLTGQ NVAIKKLSRP

                   101
Hsa_MAPK8            FQNQTHAKRA YRELVLMKCV NHKNIIGLLN VFTPQKSLEE FQDVYIVMEL MDANLCQVIQ MELDHERMSY LLYQMLCGIK HLHSAGIIHR DLKPSNIVVK
Hsa_MAPK9            FQNQTHAKRA YRELVLLKCV NHKNIISLLN VFTPQKTLEE FQDVYLVMEL MDANLCQVIH MELDHERMSY LLYQMLCGIK HLHSAGIIHR DLKPSNIVVK
Hsa_MAPK10           FQNQTHAKRA YRELVLMKCV NHKNIISLLN VFTPQKTLEE FQDVYLVMEL MDANLCQVIQ MELDHERMSY LLYQMLCGIK HLHSAGIIHR DLKPSNIVVK
Bflo_XP_035669587    ---------- ---------- ---------- ---------- -------MEL MDANLCQVIQ MDLDHERMSY LLYQMLCGIK HLHSAGIIHR DLKPSNIVVK
Bflo_XP_035669588    ---------- ---------- ---------- ---------- -------MEL MDANLCQVIQ MDLDHERMSY LLYQMLCGIK HLHSAGIIHR DLKPSNIVVK
Bflo_GETA01026605    FQNVTHAKRA YREFVLMKLV NHKNIIGLLN VFTPQKSLEE FQDVYLVMEL MDANLCQVIQ MDLDHERMSY LLYQMLCGIK HLHSAGIIHR DLKPSNIVVK
Blan_BLAG15101005    FQNVTHAKRA YREFVLMKLV NHKNIIGLLN VFTPQKSLED FQDVYLVMEL MDANLCQVIQ MDLDHERMSY LLYQMLCGIK HLHSAGIIHR DLKPSNIVVK
Blan_BL14252         FQNVTHAKRA YREFVLMKLV NHKNIIGLLN VFTPQKSLED FQDVYLVMEL M-------VMY HRPDVSLMSH APLQ------ ---------- DLKPSNIVVK
Blan_JT868948        ---------- ---------- ---------- ---------- ---------- ---------- ---------- ---------- ---------R DLKPSNIVVK
Blan_consensus       FQNVTHAKRA YREFVLMKLV NHKNIIGLLN VFTPQKSLED FQDVYLVMEL MDANLCQVIQ MDLDHERMSY LLYQMLCGIK HLHSAGIIHR DLKPSNIVVK
Bbel_XP_019639469    FQNVTHAKRA YREFVLMKLV NHKNIIGLLN VFTPQKSLEE FQDVYLVMEL MDANLCQVIQ MDLDHERMSY LLYQMLCGIK HLHSAGIIHR DLKPSNIVVK

                   201
Hsa_MAPK8            SDCTLKILDF GLARTAGTSF MMTPYVVTRY YRAPEVILGM GYKENVDLWS VGCIMGEMVC HKILFPGRDY IDQWNKVIEQ LGTPCPEFMK KLQPTVRTYV
Hsa_MAPK9            SDCTLKILDF GLARTACTNF MMTPYVVTRY YRAPEVILGM GYKENVDIWS VGCIMGELVK GCVIFQGTDH IDQWNKVIEQ LGTPSAEFMK KLQPTVRNYV
Hsa_MAPK10           SDCTLKILDF GLARTAGTSF MMTPYVVTRY YRAPEVILGM GYKENVDIWS VGCIMGEMVR HKILFPGRDY IDQWNKVIEQ LGTPCPEFMK KLQPTVRNYV
Bflo_XP_035669587    SDCTLKILDF GLARTAGTSF MMTPYVVTRY YRAPEVILGM GYTENVDVWS VGCIFGEMVR GKIVFRGNDH IDQWNKIIEQ LGTPSQDFMK RLQPTVRNYV
Bflo_XP_035669588    SDCTLKILDF GLARTAGTSF MMTPYVVTRY YRAPEVILGM GYTENVDIWS MGCICGEIVR GGVLFPGTDH IDQWNKIIEQ LGTPSQDFMK RLQPTVRNYV
Bflo_GETA01026605    SDCTLKILDF GLARTAGTSF MMTPYVVTRY YRAPEVILGM GYTENVDIWS MGCILGETVK GTVLFPGNDH IDQWNKIIEQ LGTPSQDFMK RLQPTVRNYV
Blan_BLAG15101005    SDCTLKILDF GLARTAGTSF MMTPYVVTRY YRAPEVILGM GYTENVDVWS VGCIFGEMVR GKIVFRGNDH IDQWNKIIEQ LGTPSQDFMK RLQPTVRNYV
Blan_BL14252         SDCTLKILDF GLARTAGTSF MMTPYVVTRY YRAPEVILGM GYTENVDIWS MGCILGETVK GTVLFPGTDH IDQWNKIIEQ LGTPSQDFMK RLQPTVRNYV
Blan_JT868948        SDCTLKILDF GLARTAGTSF MMTPYVVTRY YRAPEVILGM GYTENVDVWS VGCIFGEMVR GKIVFRGNDH IDQWNKIIEQ LGTPSQDFMK RLQPTVRNYV
Blan_consensus       SDCTLKILDF GLARTAGTSF MMTPYVVTRY YRAPEVILGM GYTENVDVWS VGCIFGEMVR GKIVFRGNDH IDQWNKIIEQ LGTPSQDFMK RLQPTVRNYV
Bbel_XP_019639469    SDCTLKILDF GLARTAGTSF MMTPYVVTRY YRAPEVILGM GYTENVDIWS MGCILGETVK GTVMFPGTDH IDQWNKIIEQ LGTPSQDFMK RLQPTVRNYV

                   301
Hsa_MAPK8            ENRPKYAGYS FEKLFPDVLF PADS-EHNKL KASQARDLLS KMLVIDASKR ISVDEALQHP YINVWYDPSE AEA--PPPKI PDKQLDEREH TIEEWKELIY
Hsa_MAPK9            ENRPKYPGIK FEELFPDWIF PSES-ERDKI KTSQARDLLS KMLVIDPDKR ISVDEALRHP YITVWYDPAE AEA--PPPQI YDAQLEEREH AIEEWKELIY
Hsa_MAPK10           ENRPKYAGLT FPKLFPDSLF PADS-EHNKL KASQARDLLS KMLVIDPAKR ISVDDALQHP YINVWYDPAE VEA--PPPQI YDKQLDEREH TIEEWKELIY
Bflo_XP_035669587    ENRPKYAGYS FEKLFPDVLF PAESTEHNKL KASQARDLLS KMLVIDPYKR ITVDQALQHP YVNVWYDPSE VEG--PAPIP YDHSIDEREH TVEQWKDLIY
Bflo_XP_035669588    ENRPKYAGYS FEKLFPDVLF PAESTEHNKL KASQARDLLS KMLVIDPYKR ITVDQALQHP YVNVWYDPSE VEG--PAPIP YDHSIDEREH TVEQWKDLIY
Bflo_GETA01026605    ENRPKYAGYS FEKLFPDVLF PAESTEHNKL KASQARDLLS KMLVIDPYKR ITVDQALQHP YVNVWYDPSE VEGVSPAPIP YDHSIDEREH TVEQWKDLIY
Blan_BLAG15101005    ENRPKYAGYS FEKLFPDVLF PAESTEHNKL KASQARDLLS KMLVIDPYKR ITVDQALQHP YVNVWYDPSE VEGVSPAPIP YDHSIDEREH TVEQWKDLIY
Blan_BL14252         ENRPKYAGYS FEKLFPDVLF PAESTEHNKL KASQARDLLS KMLVIDPYKR ITVDQALQHP YVNVWYDPSE VEGVSPAPIP YDHSIDEREH TVEQWKEGSE
Blan_JT868948        ENRPKYAGYS FEKLFPDVLF PAESTEHNKL KASQARDLLS KMLVIDPYKR ITVDQALQHP YVNVWYDPSE VEGVSPAPIP YDHSIDEREH TVEQWKDLIY
Blan_consensus       ENRPKYAGYS FEKLFPDVLF PAESTEHNKL KASQARDLLS KMLVIDPYKR ITVDQALQHP YVNVWYDPSE VEGVSPAPIP YDHSIDEREH TVEQWKDLIY
Bbel_XP_019639469    ENRPKYAGYS FEKLFPDVLF PAESTEHNKL KASQARDLLS KMLVIDPYKR ITVDQALQHP YVNVWYDPSE VEG--PAPIP YDHSIDEREH TVEQWKDLIY

                   401
Hsa_MAPK8            KEVMDLEERT KNGVIRGQPS PLGAAVINGS QHPSSSSSVN DVSSMSTDPT LASDTDSSLE AAAGPLGCCR -----
Hsa_MAPK9            KEVMDWEERS KNGVVKDQPS ---DAAVSSN ATPSQSSSIN DISSMSTEQT LASDTDSSLD ASTGPLEGCR -----
Hsa_MAPK10           KEVMNSEEKT KNGVVKGQPS PSGAAVNSSE SLPP-SSSVN DISSMSTDQT LASDTDSSLE ASAGPLGCCR -----
Bflo_XP_035669587    KEVVDYVQK- ---LLYGKLP LIGNSASDRS EVKLSQPQPR KRKLSQSSDD TSVCRTLDAI PAARPDATRT LTSEP
Bflo_XP_035669588    KEVVDYVQK- ---LLYGKLP LIGNSASDRS EVKLSQPQPR KRKLSQSSDD TSVCRTLDAI PAARPDATRT LTSEP
Bflo_GETA01026605    KEVVDYVQK- ---LLYGKLP LIGNSASA-- ---------- ---------- ---------- ---------- -----
Blan_BLAG15101005    KEVVDYVQK- ---LLYGKLP LIGNSASGDT CSTS------ ---------- ---------- ---------- -----
Blan_BL14252         VKISQPHPR- -----KRKLS PSPDDTKTVG TLNVGRTGSD EVKNREETSG D--------- ---------- -----
Blan_JT868948        KEVVDYVQK- ---LLYGKLP LIGNSASA-- ---------- ---------- ---------- ---------- -----
Blan_consensus       KEVVDYVQK- ---LLYGKLP LIGNSASGDT CSTS------ ---------- ---------- ---------- -----
Bbel_XP_019639469    KEVVDYVQK- ---LLYGKLP LIGNSASA-- ---------- ---------- ---------- ---------- -----
```

Supplementary 2

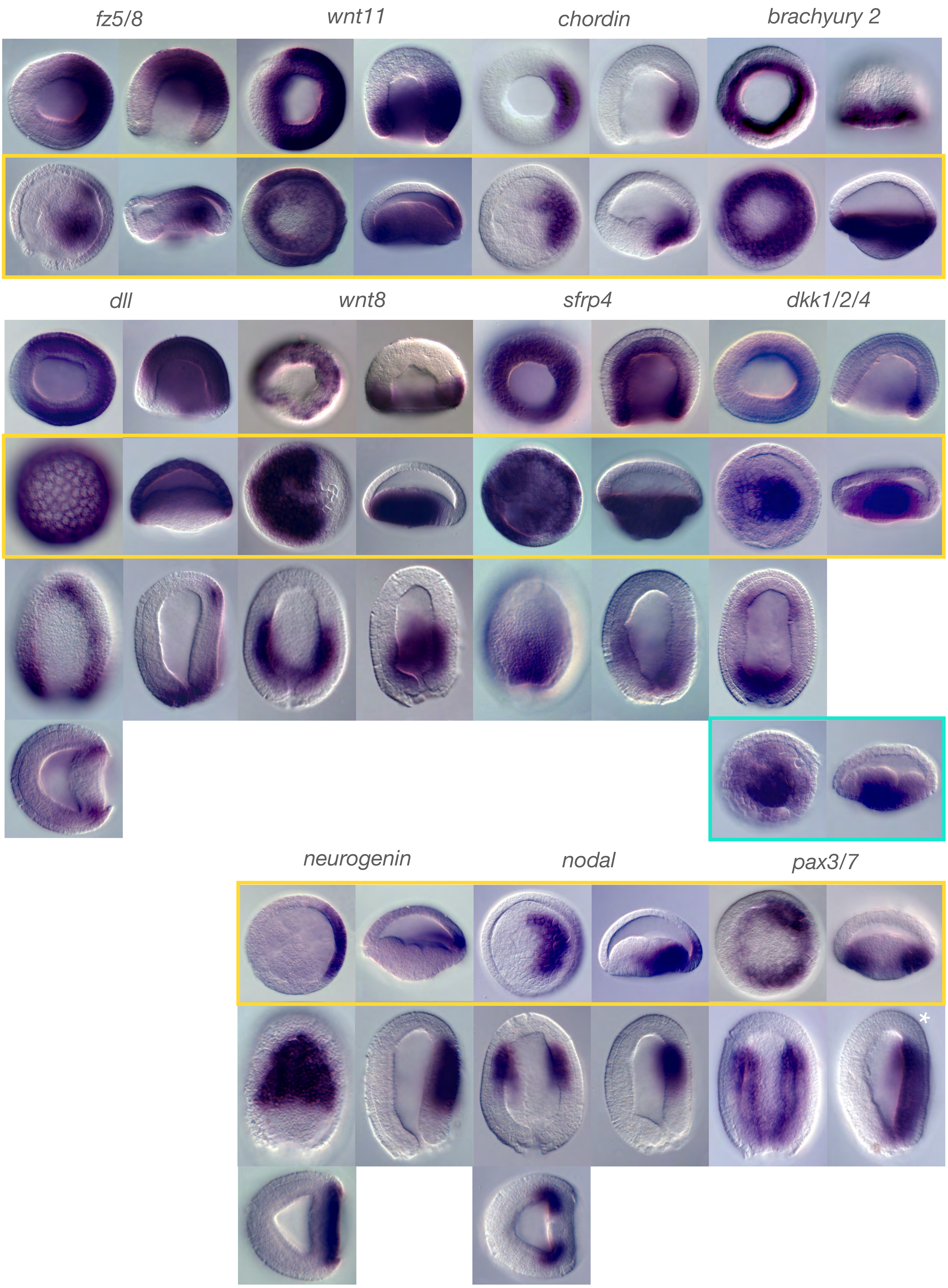

Supplementary 3

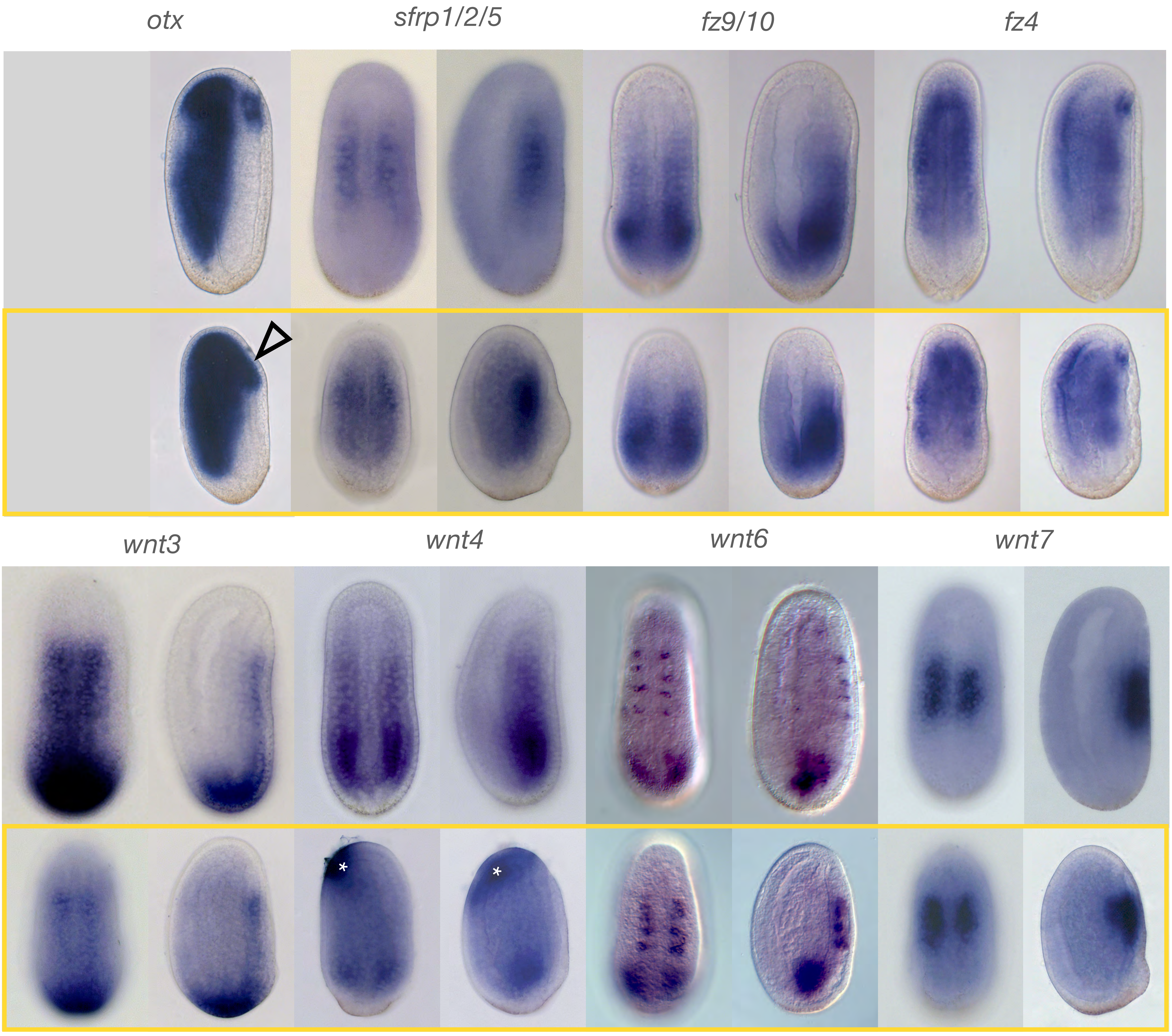

Supplementary Figure 4

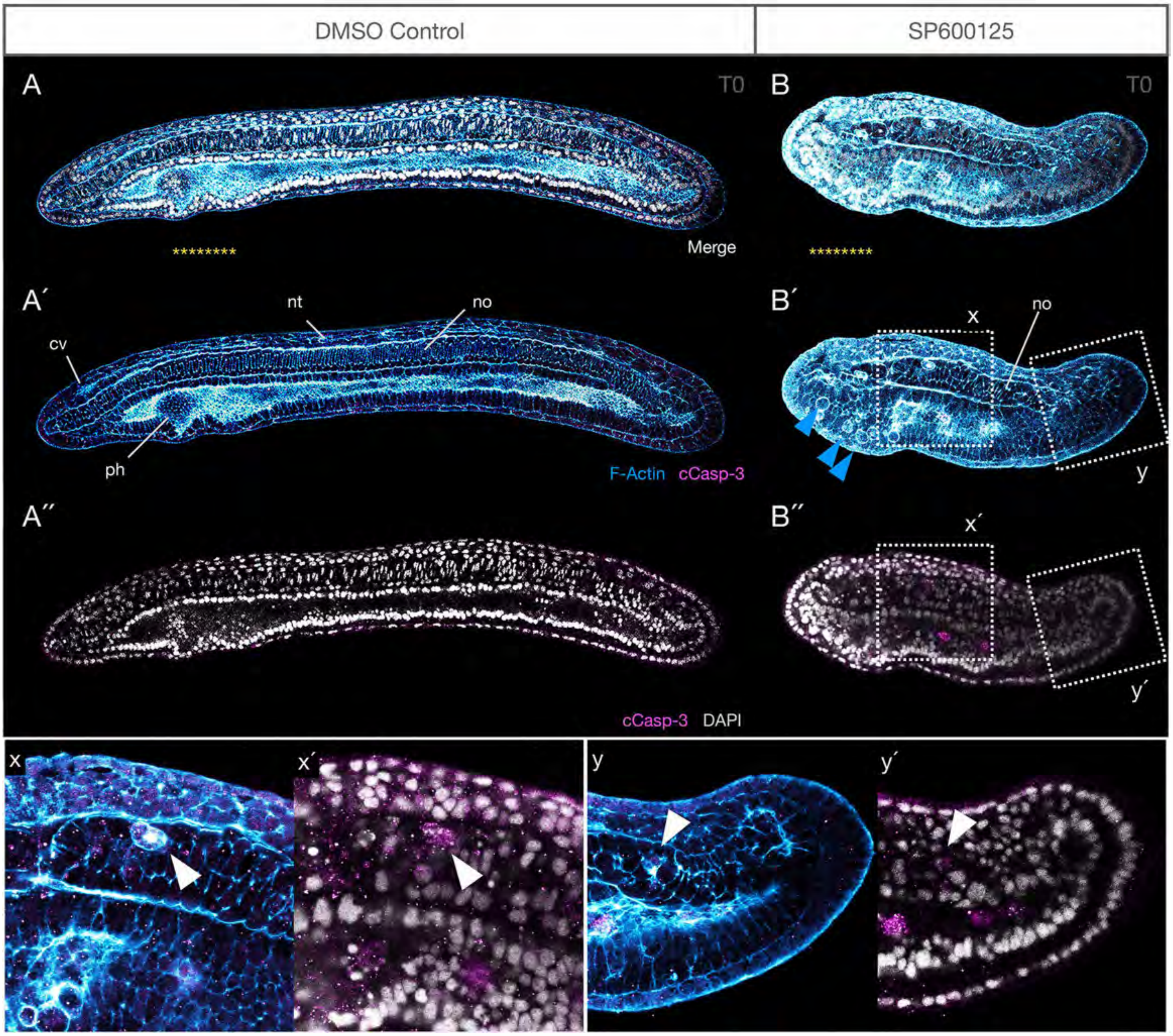

Supplement: Supplementary Figure 1 — Alignment of three JNK sequences from human (MAPK8, 9, and 10) and single orthologues from amphioxus species Branchiostoma floridae, Branchiostoma lanceolatum, and Branchiostoma belcheri. Transcriptomic data were used to manually correct errors in B. lanceolatum gene model BL14252 from Ensembl to generate a consensus sequence. The alignment was generated in Seaview using Muscle. [file Data_Sheet_2.pdf]
