# Supplementary material for: JNK Mediates Differentiation, Cell Polarity and Apoptosis During Amphioxus Development by Regulating Actin Cytoskeleton Dynamics and ERK Signalling
Source: Front Cell Dev Biol. 2021 Oct 29;9:749806. doi: 10.3389/fcell.2021.749806 (PMC8586503; doi:10.3389/fcell.2021.749806)
Supplement: Supplementary File 1 — Sequences of new probes used in this study. [file Data_Sheet_1.PDF]

Supplementary file 1

>Blan_Dkk1
ACTAGTGATTGCACCTCTGGAACAGCTCTCCGGGCACGTGCGCTTGTCGGGCCGGGTACAGGCGTC
CCTTGCGTCAGCATGGGCTTGCACACCTTGGAGAACCAGTGGCGCGCACAGCACAGCCCCTCCTGG
CAGTCGGACGACCTCAGGCACCGTGACCAAGCTGAACCTCGCTGAAACGTTTCCTTGGTGTCCTGGA
AGGTCTCTCGGCTGTCCACACTTTCAGAAGTCTCCTCCACCTCCGTCGCATCCTCTCGTTCCTCTCCGT
TGTCATGACGACGTCCCTCCAGTGCCACTCGTTCCCTGGGTCTGACGCAGATGCCGTTGAGGCAGGC
GTTGCCCTTACAGCACATGGAGTCGCGGTTGCAGCGGCGGCGGAGGCGACGACATGGCAGGCAAA
CCTTCTCGTCTCTCCGGCCGTGACAGAACTGGTCAGCATCGCATGACTCGTCGTCAAAACATCTCACC
TCAAATGTTTCTTCCTCTATCTCACGGCTGGCCTCATTCTCCGTGGTCGTCGCGTTCCTACCGACCGTC
TTGATGTAATC
>Blan_Dkk3
GATTTTCCCAACCACCAAGACTTCACCAACCATCCCCGCAACATCATGGACAGAGCCGACAACGCGA
CGGAGAACAGGACCAGGGCACTGGACACTGCGGTCCCTTGTGATACGGACAAGTCGTGCGGAGGC
GGGAAGTACTGTGACCGGCACTATGGCGTGTGCAAGCCCCGGGGACGCATGGGGGACCTGTGCCG
CCGCGACGGAAACTGCCAGGGCGGGTACGACTGCATGTTCGGCCGCTGTGAGAAGACCATCCAGG
CCGGAGAACAGGGTGCCAGGTGTAAGCACGACAAGGACTGCGGAGACAACATGTGCTGTGCGCGC
CAACACGGCGAGAGGATCTGCAAGCCGCGCCTGGCCCCCGGGCAGAAGTGCTGGGTCCCCGAGGG
AGGGCTGGACTACAGTCTGAACCAGATGTGCCCAATCACTAGT
>Blan_Fz4
AATTCACTAGTTGATGACCAAATCCACATGCTACTCGTGATCCCAACCACGAGAGACATGAAAATCTT
CAACATGTAGAGTTCCATGTTGGGTTTCGCGGAGGAATACCTCCAGAGTTCTCTATTGGAGTGTTCG
TAGAAGTAGCAGGCGATGACACAGGTAGCCGGGACGGTATGCAGCACGCTGAAGACTCCGATTTTC
ACCATCAGTCGTTCCAGTTTGTCGGTTTTCGTTCCGTCGTTCTTCATGAAGTTTCGGATGCGGAAAAG
GGAGTCAAACCCGGCTAGGAGAAAGGACGTCCCGACCACTAGGTAGGTGAACAAGGGTGCGAGCA
CGAATCCAGTCAGCGCGTCTAGATTTTGGTTCCCGACAAAACACAGTCCGGTCAGCTCGTCAGCGTC
TACCTGTTTCATGACGAAAATGACGATGGTTTTGATGGCAGGGATGGACCATGTAGCCAGGTGGAA
ATAGCTGCTGTGCATCTCTATCGCTTCATGTCCCCACTTCAGTCCAGCGGACAGGAACCAGGTGAGG
GTGAGGATGACCCACCAAATCGAGCTCGCCATGCCGAAGAAGTACAGGAACAGGAAGACGATCGC
GCAACCCGTATTTTCCAGTCCCTGTTGAATCAGAAACGCCTGTCCGTCTTCCGAGTCGCACGAGATA
GCCTTCCTTCCCACAACAAGCCTGACGATGTAGGCGATGCTGTATATGTTGTAGCACATGGAGAGAA
AGATGATGGGTCTCTCGGGATAAGCGGAATCG
>Blan_Fz5/8
GATTTGGCCTCCTGGCCCCACTTCATCCCTGCGGCCAGGAACCACGTGAAGGAGAGGATGACCCAC
CAGATGGAGCTGGCCATGCTGAAGAAGTAGATGAGCAGGAACACGATGGTGCACAGCGCCGGCCC
CGTGGTGCCGTACCGGACGATCTGTAAGTCCTTCTCGCAGGCGACCGCCTCGTGCCCCGCGATGAG
ACGGATGATGTACCCGAGGGAGACGAAGAAGTAGCAGAGCGCGAGGAAGATGATGGGTCTCTCG
GGGTACTTGAATCTGGTCATGTCTATGAGGAAGGTGGTGACGGTCATGAAGGTTGACGCACAGCAC
AGCCCGGCCCACAGCGCGATCCAGAACTTCGCGAATGTCTTTTTGCTGTTGTCAAAGTAAGGGTCGT
AGCAGGGGACCCCGCAGTTGGGCTGGTCGCCGGCAGAAATCCCGCGGTTGTACCACGACTTGGAG
CTGGGCGGGATGCTGACGAGGGGGTTGTTACAGCCGCACTTGCAGCCGCCGCTAGGCGGGGCAGG
TCTGGGCCGTCCCCCGGGGACGTCACCTGCCGTGATGGTGGGGAGGGCGGGGGTAGACTTCTGCG
TGTTTTCACCTGACGCGTTGAGAAAGTGCATACACAGCCGATCGGGGTCCCCTTGTTCGGGCAGGTC
GTCGCATTCGAACTTGTCGGGCCAGGCGAACTGGTA
>Blan_Fz1/2/7

ACTAGTGATTCTTGCAGTTGTCGATCTCCCAGGACCTCTCCCACTCCCTGCGGAACGCCTGCTCGTAG
AAGTAGCACGCGATGACGATCGTGGCGGGTACGGTGTACAGCACGCTGAAGACGCCGATCCGGAT
CATGAGTTTTTCCAGCTTGTCTGTCTTCGTCCCGTCCTGTTTGATGATCGTACGGATGCGGAACAGCG
ACACAAACCCGGCGAGAAAGAACAGCGTTCCGATCACCAGGTAGAGGAAGAGCGGCGCCAGCACG
AATCCCCGGAGAGCGTCGATGTCGGAAATGCCGACGGAGCAAACGCCGCTGAGGACGTCGCCGTCT
ACCTTCCCCATAGCGAGGATGGCGATGGTTTTGACTGCGGGGACAGCCCAAGCCGCTAGGTGGAAG
TACTGGGAGTTGGCCTCGATCGCTTCATGTCCCCACTTCATGCCTGCGGCGAGGAACCACGTTAGCG
TGAGGATGACCCACCACAGGGACGACGCCATGCTGAAGAAGTACAGCATCATGAACAGGATCGTGC
ACCCTTCCTTCTTCGTGCCCTGTGTGACCGTTGCGACGGTTTGATATCTCTGGCTGCCGTCTTGCATCC
AGACGCCTTCCATGCAGGAAACCTTTTCTTTCAGGGCATAGCCGACGATGTAGGC
>Blan_Fz9/10
ACTAGTGATTGTCCCGAAACATCCCACGAGTAGGAGGAAGGCTATCTTCACACAGTACACGGCTACC
AGAGGGATGGAGCTGGAAAGTCTACAGTCTACATTGTTGTTACAGGGGATTGCTTGGGCAGCCATG
GTCCAAGTGGACATGTTTTGTTGTTCGTAGAAGTAACAGGCGACCACGCATGTGGCTGGAACCGTG
TAGAAGACAGAAAAGATGCCGATTCGACTCATGAGTTTCTCTAGTCTTTCTGTGCTGTTTCCAGATCC
TCGCATCACTTTTCTTATCCGGAACAGGTTGACGAATCCGGCAAGGATGAAGCATGTGCCCAGGATC
AGGTAGGTGAAAAGCGGTGCGAGCACAAACCAGGTCAAAGCGGTTCGATCCTGGTTCCCGACGTA
GCAGAGTCCAGTCAGTTCGTCCCCGTCCACACGTCTCAGAGTCAAGACCACGATAGTCTTGACCGCA
GGCACTGCCCAGGCTGCCAGGTGGAAGTAGCTGCTGTGGGCTTCGATGGCTTCTTGCCCCCACTTGA
GCCCTGCTGCTAGGAACCAGGTCATAGTGAGGATAACCCACCACAGGGAGCTAGCCATGCCGAAAA
AGTACAGGATCAGGAACACAATGGTGCACCCCGTGCTTTCTAGTCCTTCCTGTATCAGGTACGGCTC
TCCTCGGTCCATGTCGCAGGAGA
>Blan_sFRP3/4
TATGACGAGGATGCCCAACCTGCTCCACCACAGCACCCAGGAGAACGCCAAACTCGCCATCGAGCA
GTACGAGAGTCTTGTGAACTTTAAGCCCAGCTGCAGCCCAGACCTGCTCCTGTTCTTCCTCTGCTCCA
TGTACGCACCCATCTGTACCCTAGATTTCATCCAGGAGCCCATCCGGCCCTGTAAGAAGGTGTGCGA
AGGTGCGCGCGCCGCCTGTGAGCCCGTGGTGCTCGCCTACAACCACACCTGGCCGGACCACCTCAA
GTGCGACGACTTACCTGTGTACGAGCTGGGCGTCTGTATCTCACCTGACGCCATCGTCACTGAAGAA
CCGCCAGAGCTCTCGGGTGGTTCGGCATCTACAGAGAGCGCAGAAATTAGGGAACACGGCAAGCC
AAGAGAAAACGGACCCCGCAAACAGAGATGCCCAAAGTGCAAACGACTGAAGGCCTCCTACAAAA
ACTACGGGAAGAAAACTTATCATTATGCGCTGAGAGTACAGGTGAAGGGTACCGAGACCCGCGGG
AGCGAGCTGGCCACGACTGTGAACGTGCTTGAGGAGTGGAAGCACCACGCCATCAGCGTGCCCAA
GGGGGAAGTGCAGCTCTGGACCAACTCCACATGTAGATGTCCCAGGCTGAAGGTGAACCAGGAGT
ACGTCATCATGGGGTACGAGGACAGCACCAATGGGCGGCTCCTGTTGCTGCCCGACACCATGGTAA
CCAACTACGCCAACAAGTGGCAAAAAAGATTCAAGAAATGGGAAAGACGGAATCACTAGT
>Blan_sFRP1/2/5
GATTCTTGCCCATGATGAGGAACTGGGACTTGGTGTCCTGGATCTCGTCACACGTGCAGACGTCGCT
GTCCTGCAGCATCAGGGTCAGGTTGCGCATGTCACGTTTCCGGATCCCAGTCTTCTTCATGACGCGTT
TGCGACGGCCCACCACGATTTTAGTGTCTCTGCGGGTGATCTTGATCTGCTCGATCTTCGCTCTTATA
ACGAAGTCATTTTGGCAGTAGTACCCAACCAGATCCTTCCAGCCCTCTCCGTTGCTCTGACAGGCCAT
GCACACTGGCTGGGGCTGATCCACCTCAGGTGGGAGAGGATCCACTTCCCCGGACTGCGTGGTGAT
GCAGAGGTCGTTGTCCGGGGGAAACTTGTCACACCTGAGCATATCCGGCCACGGGAAGCCAAACTT
CAACATGACCGGAGAACAGCCGTCGCGGACGGCCTCGCACAGGGAGCGGCAGGGGTAGATTGGAT
GATCCAAACACACTGGGGCAAACAGAGAGCACAGAAATAAACGGGTGTTTGGGTGGCACCGTTTA
GGCAACAGTGGGACCCAGGTACCGGCCTGGTCCAACACTTCGGTCATTGTCTCGTGGCCTAAGAGG
TTGGGAAGTCTCATTTGCGAGTACGCAATGTTTCTGCACAGAGTCAGCTCCGAGGGAATAATCACT
